# Excitation Energy Transfer in an Intermediate Regime: A Multiconfigurational Gaussian Wavepacket Study of a Light-Harvesting Supramolecular Dyad

**DOI:** 10.1021/acs.jpclett.6c00100

**Published:** 2026-03-09

**Authors:** Sreeja Loho Choudhury, Maximiliane Horz, Rainer Hegger, Rocco Martinazzo, Irene Burghardt

**Affiliations:** † Institute of Physical and Theoretical Chemistry, 9173 Goethe University Frankfurt, Max-von-Laue-Str. 7, 60438 Frankfurt, Germany; ‡ Department of Chemistry, 9304 Università degli Studi di Milano, Via Golgi 19, 20133 Milano, Italy

## Abstract

Ultrafast excitation energy transfer (EET) is studied for a supramolecular rhodamine-BODIPY dyad, which exemplifies EET systems that fall into a non-Förster regime where coherent effects are important. A key question that arises concerns the transition between coherent and kinetic transfer regimes, which is addressed here based on real-time quantum dynamics and the time-evolving state-to-state flux that transitions from early time transients to a quasi-stationary regime. Multiconfigurational wavepacket calculations are carried out using the two-layer Gaussian-based multiconfiguration time-dependent Hartree (2L-GMCTDH) method, in conjunction with the thermofield dynamics method in order to include thermalization of low-frequency modes. Several characteristic time scales are identified that are intimately connected to the flux evolution and decoherence phenomena. An almost fully decoherent state is reached at around 75 fs, but the purity is restored to a large extent as the transfer to the acceptor state proceeds. It is found that the ultrafast EET step that is almost complete at around 200 fs is mediated by vibronic resonance effects, which lead to an athermal nonequilibrium state of the donor moiety, exhibiting mode-selective vibrational excitation following the EET transfer. A slower time scale associated with a kinetic regime shows a non-negligible temperature dependence.

## Graphical abstract

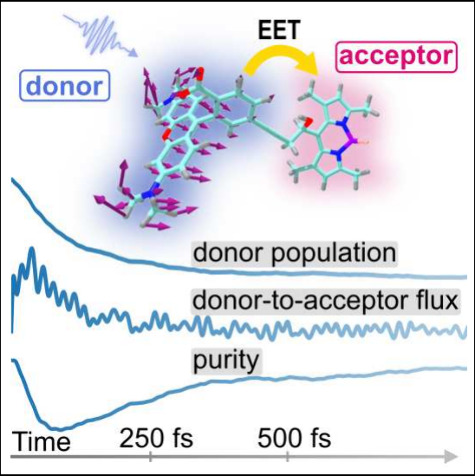

Energy and charge transfer in photoexcited molecular systems often fall into an intermediate regime where rate theories are not applicable.^1–3^ While oscillatory transients are a landmark of electronic and/or vibrational coherence, some systems exhibit coherent transients accompanied by near-exponential behavior and monotonic population decay due to efficient nonadiabatic transitions on ultrafast conversion time scales. In these cases, multiple decay time scales exist and initial nonequilibrium evolution transitions seamlessly to a kinetic regime. If a separation of time scales is given within a system-bath type treatment, it has been recognized^4^ that kinetic equations can be constructed from a nonequilibrium flux at a plateau time, rather than computing equilibrium time correlation functions. In the absence of such a time scale separation, i.e., in a non-Markovian regime,^1,5,6^ the situation is more complicated. The question then arises how exactly the transition to a kinetic regime can be identified, especially if the initial transients extend over a significant part of the experimental observation window. Furthermore, the role of decoherence and energy dissipation, and the role of specific modes in these processes, are crucial in determining the relevant time scales.

In the present study, we investigate a molecular donor–acceptor dyad exhibiting ultrafast excitation energy transfer (EET).^7^ The polyatomic dyad system, shown in Figure 1, exemplifies the coupling of the electronic conversion process to a high-dimensional vibrational bath with a structured spectral density (SD).^1,5^ In an earlier combined experimental and theoretical study addressing this dyad,^7^ a highly efficient EET process on a sub-500 fs scale was found, not compatible with Förster theory.^1,2^ The context of these investigations was the quest for modular architectures for efficient photoinduced uncaging, where the acceptor is a photoremovable protecting group (PPG) that undergoes cleavage subsequent to the energy transfer step. The theoretical analysis of ref 7, carried out by some of us, was based on a first-principles parametrized linear vibronic coupling (LVC) model^1,8^ in conjunction with high-dimensional quantum dynamical simulations using the multilayer multiconfiguration time-dependent Hartree (ML-MCTDH) method.^9–11^ An ultrafast, monotonic population evolution was found, exhibiting an initial time scale of ca. 200 fs where a significant electronic flux was observed. At later times, the population transfer slows down, accompanied by a weaker, oscillatory flux. These observations are suggestive of a system which lies at the border between a coherent and a kinetic regime.

**Figure 1:**
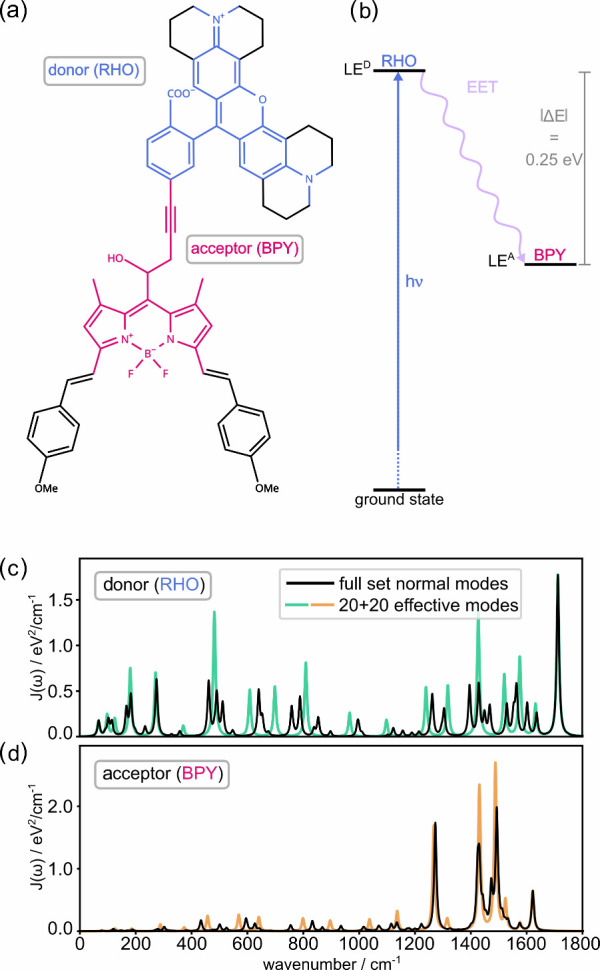
a) Molecular structure of the rhodamine-BODIPY (RHO-BPY) dyad, with the RHO donor moiety labeled in blue and the BPY acceptor moiety labeled in magenta. Due to the alkyne linker, electronic excitations of the two fragments are essentially decoupled.^7^ b) Schematic representation of the photoexcitation to the locally excited donor state |LE^*D*^⟩, followed by EET to the locally excited acceptor state |LE^*A*^⟩. c) Donor spectral density with the full set of *N*_*D*_^(0)^ = 121 normal modes in the relevant frequency range, as compared with the reduced set of *N*_*D*_ = 20 effective modes. d) Likewise, acceptor spectral density with the full set of *N*_*A*_^(0)^ = 93 normal modes as compared with the reduced set of *N*_*A*_ = 20 effective modes. All SDs are convoluted with a Lorentzian line shape function with a full width at half-maximum (fwhm) of 5 cm^–1^. See Sec. S1 in the Supporting Information for further details.

The goal of the present study is therefore to unravel how decoherence and dissipation play out for the structured SD of this system, how the time evolution is reflected in the nonequilibrium flux and the electronic purity, and to what extent mode specificity is of importance. Our findings show a multiple-time-scale process with (i) an initial time scale of <5 fs determined by pure electronic evolution, followed by (ii) vibronic decoherence leading to an almost fully decoherent state at 75 fs, (iii) a distinct nonstationary coherent flux during a ∼150 fs time scale which determines the major part of the EET transfer process, (iv) followed by a transition to a kinetic regime with slower decay and a small but non-negligible temperature dependence. Furthermore, we find that the initial 100 fs of the system’s evolution are quite well represented by just two effective modes,^12–16^ which induce 80% of the population transfer. Crucially, ultrafast and efficient transfer necessitates resonant vibrational modes in the lower-frequency range, besides the high-frequency modes that drive the initial nonadiabatic crossings.

Beyond the interest of clarifying the EET mechanism in the present system, a detailed understanding of EET in an intermediate regime is essential for guiding the rational design and optimization of various related functional donor–acceptor architectures.

## EET Hamiltonian and Effective-Mode Representation

The LVC model developed in ref 7 refers to the full normal mode space of the supramolecular dyad, comprising 267 normal modes. Due to the rigid alkyne bridge between the donor (*D*) and acceptor (*A*) fragments (see Figure 1a), the normal modes separate into two subsets, associated with the *D* vs *A* fragments, respectively. In ref 7, ML-MCTDH studies were carried out for this model, where one of the torsional normal modes on the rhodamine (RHO) fragment was discarded due to its nonbonding potential. In the present study, we further omit a number of weakly coupled high-frequency modes above 3000 cm^–1^ and low-frequency modes below 50 cm^–1^, resulting in *N*_*D*_^(0)^ = 121 donor modes and *N*_*A*_^(0)^ = 93 acceptor modes (see Sec. S1 in the Supporting Information). In the following, we show that a much lower-dimensional variant of the model, with a total of 40 effective modes, with *N*_*D*_ = *N*_*A*_ = 20, is able to capture the salient features of the dynamics.

The LVC Hamiltonian reads as follows:

1 $${Ĥ}^{\mathrm{LVC}}={Ĥ}^{\mathrm{el}}+{Ĥ}^{\mathrm{vib}}+{Ĥ}^{\mathrm{el‐}\mathrm{vib}}$$

with the electronic part

$${Ĥ}^{\mathrm{el}}=\Delta E\;\left|{\mathrm{LE}}^{A}\right\rangle \left\langle {\mathrm{LE}}^{A}\right|+\gamma_{DA}\left(\left|{\mathrm{LE}}^{D}\right\rangle \left\langle {\mathrm{LE}}^{A}\right|+\mathrm{c.c.}\right)$$ 2

where *ΔE* = −0.25 eV is the donor–acceptor offset, and γ_*DA*_ = 0.024 eV is the EET coupling between the two locally excited (LE) states, which was computed by a transition density approach (neglecting exchange effects).^7^ Since the EET coupling is coordinate-independent, the Hamiltonian belongs more specifically to the class of spin-boson models,^1,5,6^ which do not exhibit conical intersection topologies.^8^ The LE states are Frenkel-type exciton states^1,17^ which span the combined donor/acceptor Hilbert space and correspond to |LE^*D*^⟩ = |*S*_1_^RHO^⟩ ⊗ |*S*_0_^BPY^⟩ and |LE^*A*^⟩ = |*S*_0_^RHO^⟩ ⊗ |*S*_1_^BPY^⟩, respectively, in terms of the ground states (*S*_0_) and singly excited states (*S*_1_) of the isolated donor (RHO) and acceptor (BPY) species.^7^

The vibrational and vibrational-electronic (vibronic) parts of the Hamiltonian eq 1 are given as a sum of contributions belonging to the two |LE^*s*^⟩ states, with *s* = *D*, *A*,

3 $${Ĥ}^{\mathrm{vib}}=\underset{s=D,A}{\sum }\overset{N_{s}}{\underset{n=1}{\sum }}\frac{\omega_{n,s}}{2}\left({\mathrm{x̂}}_{n,s}^{2}+{\mathrm{p̂}}_{n,s}^{2}\right)1$$

and

4 $${Ĥ}^{\mathrm{el‐}\mathrm{vib}}=\underset{s=D,A}{\sum }\overset{N_{s}}{\underset{n=1}{\sum }}\kappa_{n,s}{\mathrm{x̂}}_{n,s}\left|{\mathrm{LE}}^{s}\right\rangle \left\langle {\mathrm{LE}}^{s}\right|$$

where {ω_*n,s*_} are the mode frequencies, {κ_*n,s*_} are state-specific vibronic couplings, and mass- and frequency-weighted coordinates are used throughout. The electronic ground state of the supermolecular system is not explicitly included in the model, but it determines the initial conditions, here {⟨*x*_*n,s*_⟩(*t* = 0) = 0}.

As mentioned above, effective-mode reduction techniques^12–16^ are used to obtain *N*_*D*_ = *N*_*A*_ = 20 effective modes, whose vibronic couplings subsume the couplings of the original system. To this end, orthogonal transformations are employed, which initially combine the vibronic couplings into two effective modes while casting the Hessian matrix of the residual modes into a band-diagonal form. This is followed by truncation at a chosen order in the residual space, along with rediagonalization in the bath subspace.^16^ This procedure is further detailed in Sec. S1 in the Supporting Information, where the convergence is also illustrated.

The resulting discrete spectral densities for the *D* vs *A* subspaces read as follows:

$$J_{D}\left(\omega \right)=\frac{\pi }{2}\overset{N_{D}}{\underset{n=1}{\sum }}\kappa_{n,D}^{2}\delta (\omega -\omega_{n,D})\;;\;J_{A}\left(\omega \right)=\frac{\pi }{2}\overset{N_{A}}{\underset{n=1}{\sum }}\kappa_{n,A}^{2}\delta \left(\omega -\omega_{n,A}\right)$$ 5

In Figure 1c-d, a continuous version of the SD’s is shown following convolution with a Lorentzian line shape function. At the level of the present *N*_*D*_ = *N*_*A*_ = 20 mode representation, the agreement with the original SD is good, even though not perfect (noting that, e.g., *N*_*D*_ = *N*_*A*_ = 40 modes bring significantly closer agreement with the original SD as shown in Figure S2 in the Supporting Information). Even so, the smaller model with *N*_*D*_ = *N*_*A*_ = 20 will be shown to reproduce the key features of the EET dynamics. At this level of treatment, the reorganization energies for the two fragments are given as λ_*D*_ = ∑_*n*_^*N*_*D*_^ (κ_*n,D*_)^2^/(2ω_*n,D*_) = 0.068 eV and λ_*A*_ = ∑_*n*_^*N*_*A*_^ (κ_*n,A*_)^2^/(2ω_*n,A*_) = 0.046 eV.

While the EET process proceeds downhill, the frequency range of the SD and the reorganization energies lie significantly below the electronic energy gap of |*ΔE*| = 0.25 eV, such that the vibrational environment is essentially off-resonant. Even though weakly coupled CH and OH modes exist in a higher frequency range (>3000 cm^–1^, or 0.37 eV), these do not meet a resonance condition either and are found to play a negligible role in the dynamics;^7^ hence, these modes have been discarded in the effective-mode construction as mentioned above (see Sec. S1.1 in the Supporting Information). As a result, resonant *D*-*A* transfer will be seen to necessitate nonlinear interactions involving two or more vibrational modes.

Figure 2 illustrates some of the relevant effective modes localized on the *D* vs *A* moieties. The full set of 40 modes are shown in Figure S3 in the Supporting Information. Based on the effective-mode transformations, these modes are linear combinations of normal modes, which are, in turn, representable in terms of mass-weighted Cartesian displacements. Normal modes were obtained as described in ref 7, from the Hessian based on TDDFT calculations. Details of the relevant electronic structure calculations are given in the Supporting Information of ref 7.

**Figure 2:**
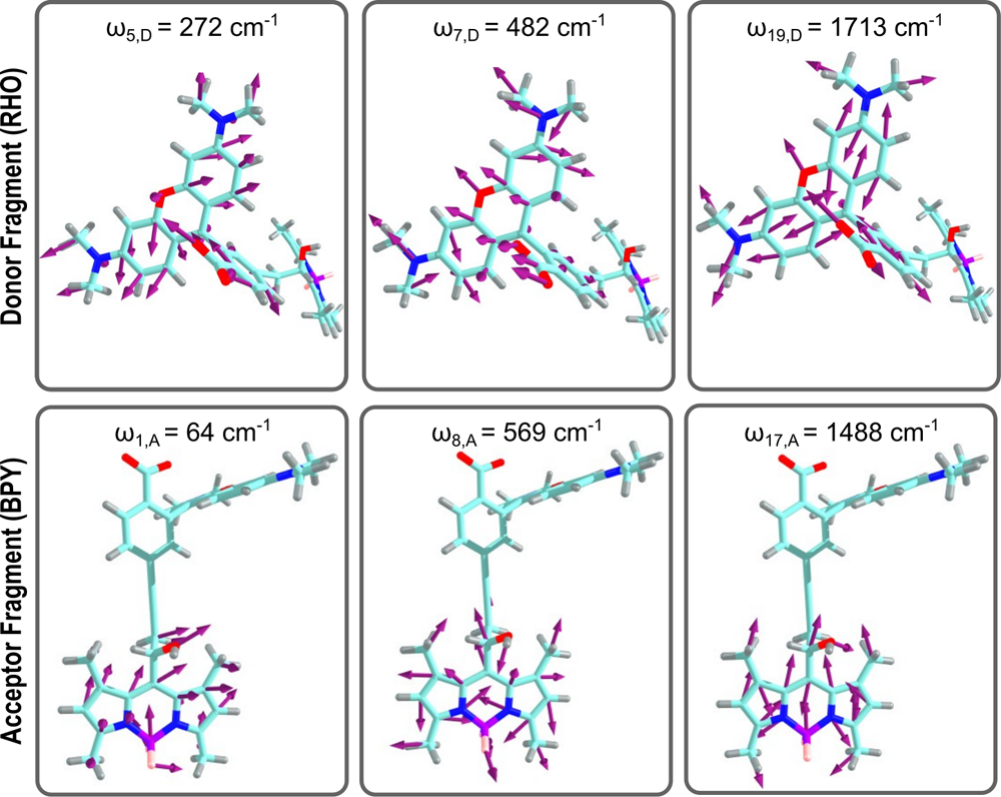
Representative effective modes localized on the donor vs acceptor fragments. The effective modes are generated by orthogonal coordinate transformations in the normal mode space and can again be represented in terms of mass-weighted Cartesian displacements.

## Thermofield Hamiltonian

The above Hamiltonian will be augmented by fictitious modes within the thermofield dynamics (TFD) approach,^18,19^ which are added to a subset of low-frequency vibrations that are susceptible to thermal excitation. The TFD approach maps a thermal equilibrium state onto a pure state in a duplicated Hilbert space where each physical mode is accompanied by a fictitious twin mode. In practice, this allows one to carry out pure-state wavefunction simulations in just the same way as for a zero-temperature system, except that additional degrees of freedom are included and temperature-dependent couplings will be introduced as explained below.

For example, for a harmonic oscillator, an auxiliary oscillator is introduced, denoted by a tilde symbol, such that a two-mode vibrational ground state reads |φ_0_, φ̃_0_⟩ = |φ_0_⟩ ⊗ |φ̃_0_⟩. A thermal wavefunction representing the mixed state of the physical mode is created from the two-mode vibrational ground state by the action of a thermalizing operator *T̂*(θ):^18,19^

6 $$\left|\psi_{T}\right\rangle =\mathrm{T̂}\left(\theta \right)\left|\varphi_{0},{\mathrm{\varphi ̃}}_{0}\right\rangle \;\mathrm{where}\;\mathrm{T̂}\left(\theta \right)=e^{-iĜ\left(\theta \right)}$$

where *Ĝ*(θ) is the generator of a so-called Bogoliubov transformation^18,19^

7 Ĝ(θ)=−θ(x̂p̃−p̂x̃)

where bilinear coordinate-momentum couplings appear between the physical operators *x̂* and *p̂* = −*i*∂/∂*x* and the tilde operators *x̃* and *p̃* = *i*∂/∂*x̃*. The mixing parameter θ = arctanh­(exp­(−ω/(2*k*_*B*_ *T*))) depends on the ratio between the oscillator frequency and the thermal energy, with θ → 0 as temperature tends to *T* = 0 K. Hence, the Bogoliubov transformation involves temperature-dependent mixing between the coordinate and momentum variables of the physical and tilde spaces, generating a so-called two-mode squeezed vacuum state.^20^ The thermal state eq 6 evolves under the thermofield Hamiltonian *Ĥ*_*T*_(*x̂*, *p̂*; *x̃*, *p̃*) = *Ĥ*(*x̂*, *p̂*) – *H̃*(*x̃*, *p̃*), where *H̃* is the so-called tildian operator which defines the evolution in the tilde subspace. The tildian is usually defined as *H̃* = *Ĥ* for a Hermitian Hamiltonian, even though the choice is arbitrary to start with.^18,19^ With this choice, *Ĥ*_*T*_ induces symmetric motion in the real and tilde subspaces, with the tilde subspace evolving backward in time.

While the thermalizing transformation acts on the wavefunction in eq 6, an alternative formulation moves the transformation from the wavefunction to the Hamiltonian:^19,21,22^

$${Ĥ}_{T}^{\theta }={\mathrm{T̂}}^{†}\left(\theta \right){Ĥ}_{T}\mathrm{T̂}\left(\theta \right)={\mathrm{T̂}}^{†}\left(\theta \right)\left(Ĥ-\mathrm{H̃}\right)\mathrm{T̂}\left(\theta \right)$$ 8

As a result of this inverse Bogoliubov transformation (iBT), *Ĥ*_*T*_^θ^ acts on the zero-temperature state |φ_0_, φ̃_0_⟩, which represents a significant advantage for quantum dynamical calculations.^21,23–25^

In cases where the iBT Hamiltonian eq 8 can be determined analytically, this variant of TFD is therefore highly convenient. This is the case for the LVC Hamiltonian, as pointed out in refs 21 and 22. For subsets of *N*_*s*_^*T*^ modes which are thermalized per fragment *s*, the vibrational and vibronic parts of the Hamiltonian read as follows:

9 $${Ĥ}_{T}^{\mathrm{vib}}=\underset{s=D,A}{\sum }\overset{N_{s}^{T}}{\underset{n=1}{\sum }}\frac{\omega_{n,s}}{2}\left({\mathrm{x̂}}_{n,s}^{2}+{\mathrm{p̂}}_{n,s}^{2}-{\mathrm{x̃}}_{n,s}^{2}-{\mathrm{p̃}}_{n,s}^{2}\right)1$$

and

10 $${Ĥ}_{T}^{\mathrm{el‐}\mathrm{vib}}=\underset{s=D,A}{\sum }\overset{N_{s}^{T}}{\underset{n=1}{\sum }}\left(\kappa_{n,s}\;\cosh\left(\theta_{n}\right){\mathrm{x̂}}_{n,s}+\kappa_{n,s}\;\sinh\left(\theta_{n}\right){\mathrm{x̃}}_{n,s}\right)\left|{\mathrm{LE}}^{s}\right\rangle \left\langle {\mathrm{LE}}^{s}\right|$$

where the effective iBT/TFD vibronic couplings κ_*n,s*_^*T*^ = κ_*n,s*_ cosh­(θ_*n*_) and κ̃_*n,s*_^*T*^ = κ_*n,s*_ sinh­(θ_*n*_) arise for the real vs auxiliary (tilde) modes.

When combined with the electronic Hamiltonian and the larger subset of nonthermalized (but dynamically active) vibrations, the LVC Hamiltonian employed in the present study reads as follows:

11 $${Ĥ}_{T}^{\mathrm{LVC}}={Ĥ}^{\mathrm{el}}+\left({Ĥ}_{0}^{\mathrm{vib}}+{Ĥ}_{0}^{\mathrm{el‐}\mathrm{vib}}\right)+\left({Ĥ}_{T}^{\mathrm{vib}}+{Ĥ}_{T}^{\mathrm{el‐}\mathrm{vib}}\right)$$

where *Ĥ* ^el^ is defined in eq 2 and

12 $${Ĥ}_{0}^{\mathrm{vib}}+{Ĥ}_{0}^{\mathrm{el‐}\mathrm{vib}}=\underset{s=D,A}{\sum }\overset{N_{s}}{\underset{n=N_{s}^{T}+1}{\sum }}\left(\frac{\omega_{n,s}}{2}\left({\mathrm{x̂}}_{n,s}^{2}+{\mathrm{p̂}}_{n,s}^{2}\right)1+\kappa_{n,s}{\mathrm{x̂}}_{n,s}\left|{\mathrm{LE}}^{s}\right\rangle \left\langle {\mathrm{LE}}^{s}\right|\right)$$

describes the set of nonthermalized modes while the thermalized modes are described according to eqs 9 and 10. In the present study, only modes with frequencies ω_*n,s*_ ≤ *k*_*B*_ *T* are thermalized, such that *N*_*s*_^*T*^ = 4 per fragment and *N*_*s*_ – *N*_*s*_^*T*^ = 16. Hence, a total number of 48 vibrational modes (i.e., 40 real modes and 8 tilde modes) are propagated, to account for the thermal distribution of the low-frequency bath modes.

## 2L-GMCTDH Calculations

Quantum dynamical calculations based on the vibronic coupling Hamiltonian eq 11 including thermalization of selected modes are carried out using the two-layer Gaussian-based multiconfiguration time-dependent Hartree (2L-GMCTDH) method.^26–29^ This method was introduced in ref 26 in order to improve on the flexibility of moving coherent state basis sets. A flexible first layer composed of orthogonal single-particle functions (SPFs) is introduced, where each SPF is expressed as a superposition of Gaussian wavepackets (GWPs) of fixed width in the second layer. This method is the simplest version of a multilayer (ML-GMCTDH) approach,^26,29^ where (*M* – 1) SPF layers are combined with a final GWP-based layer. In the present work, an in-house code developed in refs 27 and 28 is employed.

For systems composed of several electronic states, different variants were explored in ref 28. Anticipating that the dynamics in the dyad system is strongly state-dependent, we use here state-specific basis sets, corresponding to the so-called multiset approach.^9,28^ From this vantage point, decoherence effects appear as loss of overlap of Gaussian wavepackets evolving on different potential surfaces.^6,30^

The multiset 2L-GMCTDH wavefunction reads as follows:

$$\left|\Psi \left(x,t\right)\right\rangle =\overset{n_{s}}{\underset{s}{\sum }}\underset{J}{\sum }A_{J}^{\left(s\right)}\left(t\right)\Phi_{J}^{\left(s\right)}\left(x,t\right)\left|s\right\rangle =\overset{n_{s}}{\underset{s}{\sum }}\underset{J}{\sum }A_{J}^{\left(s\right)}\left(t\right)\overset{f^{\left(s\right)}}{\underset{\kappa =1}{\prod }}\chi_{j_{\kappa }}^{\left(\kappa ,s\right)}\left(x_{\kappa },t\right)\left|s\right\rangle$$ 13

for *n*_*s*_ electronic states |*s*⟩ and state-dependent configurations Φ_*J*_^(*s*)^, with the following decomposition of state-dependent first-layer SPFs χ_*j*_^(κ,*s*)^ into state-dependent second-layer GWPs:

$$\chi_{j}^{\left(\kappa ,s\right)}\left(x_{\kappa },t\right)=\underset{L}{\sum }B_{j,L}^{\left(\kappa ,s\right)}\left(t\right)G_{L}^{\left(\kappa ,s\right)}\left(x_{\kappa },t\right)=\underset{L}{\sum }B_{j,L}^{\left(\kappa ,s\right)}\left(t\right)\overset{f^{\left(\kappa ,s\right)}}{\underset{\mu =1}{\prod }}g_{l_{\mu }}^{\left(\kappa ,\mu ,s\right)}\left(x_{\kappa ,\mu },t\right)$$ 14

In the present study, the first and second-layer partitioning is assumed to be the same for both electronic states, such that *f* ^(*s*)^ = *f* and *f* ^(κ,*s*)^ = *f* ^(κ)^. The first-layer and second-layer coefficients as well as the GWP parameters evolve under fully variational equations of motion, as described in ref 26. Further details and numerical aspects are summarized in Sec. S2 in the Supporting Information. Figure 3 depicts the two-layer structure of the wavefunction employed in the present calculations.

**Figure 3:**
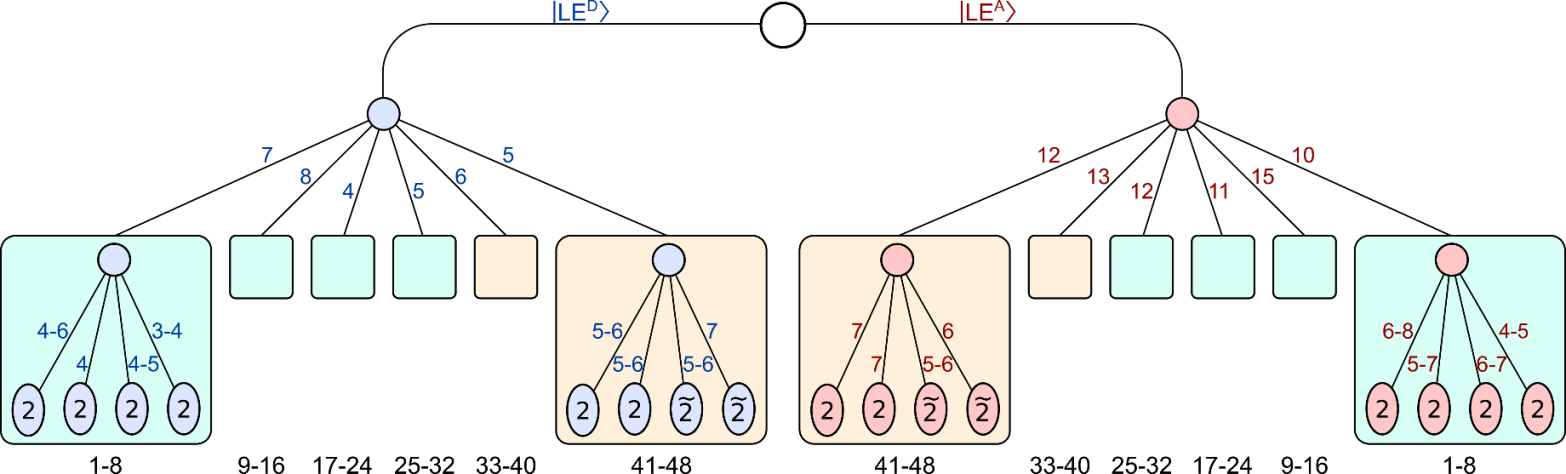
Graphical representation of the 2L-GMCTDH wavefunction. Two separate, parallel branches are constructed in a so-called multiset scheme, where each vibrational mode (labeled from 1 to 48 at the bottom of the schematic) appears in both branches, pertaining to the |LE^*D*^⟩ and |LE^*A*^⟩ states, respectively. Each branch consists of two layers, with the first layer organized into 6 orthogonal single-particle functions, each of which is represented by 4 second-layer GWPs. Each GWP consists of two modes, which are either of real or tilde type; that is, 20 GWPs represent 40 real modes, while 4 GWPs represent 8 tilde modes. Overall, 6 first-layer SPFs are split into 24 GWPs representing 48 modes. Each first-layer SPF and its decomposition into GWPs is indicated by colored boxes, where beige-colored boxes include thermalized real/tilde GWP pairs, whereas turquoise boxes consist of real GWPs. The numbers indicated alongside the edges indicate the number of SPFs vs GWPs in the |LE^*D*^⟩ state (colored in blue) and in the |LE^*A*^⟩ state (colored in red).

Based on the above wavefunction representation, the reduced electronic density matrix reads as follows:

$${\mathrm{\rho ̂}}^{\mathrm{el}}\left(t\right)=\underset{ss^{\prime }}{\sum }\left(\underset{JJ^{\prime }}{\sum }A_{J\prime }^{\left(s\prime \right)*}\left(t\right)A_{J}^{\left(s\right)}\left(t\right)\left\langle \Phi_{J\prime }^{\left(s\prime \right)}\left(t\right)\right|\Phi_{J}^{\left(s\right)}\left(t\right)\rangle \right)\left|s\right\rangle \left\langle s^{\prime }\right|=\underset{ss^{\prime }}{\sum }\rho_{ss\prime }^{\mathrm{el}}\left(t\right)\left|s\right\rangle \left\langle s^{\prime }\right|$$ 15

We note that the configurations belonging to two different electronic states are not orthogonal to each other, such that the relevant overlap integrals need to be computed, ⟨Φ_*J*′_^(*s^′^*)^|Φ_*J*_^(*s*)^⟩ = ∏_κ_ ⟨ $\chi_{j_{\kappa }^{\prime }}^{\left(\kappa ,s^{\prime }\right)}$ |χ_*j*_κ__^(κ,*s*)^⟩. These contain, in turn, GWP overlap integrals according to the decomposition of first-layer SPF functions into GWPs given in eq 14. Hence, the loss of GWP overlap encodes decoherence effects in the present description, as mentioned above.

Several quantities are derived from the reduced electronic density matrix, notably the purity, which provides a measure of the mixed-state character of the time-evolving state:^6^

16 $$P\left(t\right)={\mathrm{Tr}}_{\mathrm{el}}\left\{({\mathrm{\rho ̂}}^{\mathrm{el}}\left(t\right){)}^{2}\right\}$$

where Tr_el_ refers to the trace over the electronic subspace. Further, the transient state-to-state flux relates to the imaginary part of the electronic coherence^4,31–33^

17 jss′(t)=2γDAIm(ρss′el(t))

for *s* ≠ *s*′. The transient flux defines the rate of change of the electronic state populations, see the Appendix for derivation of the relevant continuity equation,

18 $${\mathrm{\rho ̇}}_{ss}^{\mathrm{el}}=\sum_{s\prime }j_{s\prime s}(t)$$

with *s*′ ≠ *s*.

Finally, we will refer to a representation of the reduced electronic density matrix in terms of Pauli matrices,

19 $${\mathrm{\rho ̂}}^{\mathrm{el}}=\frac{1}{2}(s_{x}{\mathrm{\sigma ̂}}_{x}+s_{y}{\mathrm{\sigma ̂}}_{y}+s_{z}{\mathrm{\sigma ̂}}_{z}+s_{0}1̂)$$

with *s*_0_ = 1 and

20 sx=ρ12el+ρ21el=2Reρ12el(20a)sy=i(ρ12el−ρ21el)=−2Im⁡ρ12el(20b)sz=ρ22el−ρ11el(20c)

which can be conveniently represented as a vector ***s*** = (*s*_*x*_, *s*_*y*_, *s*_*z*_) on the Bloch sphere.^6^ In general, the length of the vector is ∥ ***s*** ∥ ≤ 1, with ∥ ***s*** ∥ = 1 if and only if the system is in a pure state. For mixed states, the vector therefore lies inside the Bloch sphere.

## EET Dynamics

Figure 4a shows the observed decay of the donor population and the concomitant rise of the acceptor population. While the decay is monotonic, it contains several time scales that are connected to the time evolution of the electronic flux of eq 17 according to the continuity equation (eq 18) detailed in the Appendix. The time-evolving flux is shown in Figure 4b, while the real part of the electronic coherence is shown in Figure 4c. The evolution of these components of the electronic density matrix is also reflected in the purity shown in Figure 4d. While these components depend on the diabatic representation that is chosen, e.g., localized vs delocalized exciton states, the localized basis is naturally adapted to the transfer dynamics in the present system, where the RHO donor is initially excited.^7^

**Figure 4:**
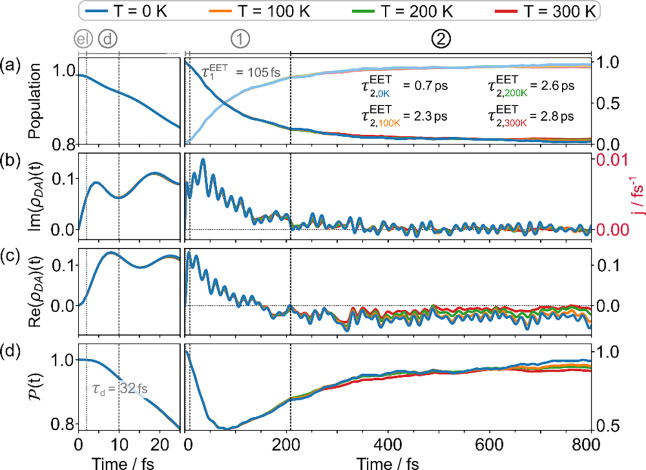
EET dynamics of the donor–acceptor dyad at different temperatures (*T* = 0, 100, 200, 300 K). The l.h.s. panels show a zoom-in on the initial 25 fs interval, while the r.h.s. panels show the evolution during the full simulation interval of 800 fs. a) Population decay on the donor and concomitant rise on the acceptor. b) Imaginary part of the electronic coherence and related scale for the flux *j* = *j*_*DA*_ indicated on the r.h.s. in red. c) Real part of the electronic coherence. d) Purity of the electronic subsystem. Several characteristic intervals within the EET process are marked by dashed vertical lines: notably, in the l.h.s. panel, the electronic time scale τ_el_ ∼ 2 fs and a time scale of ∼10 fs where a Gaussian purity decay holds, and in the main panel on the r.h.s., the time window ∼200 fs where coherent EET is observed. The indicated time constants are discussed in the text.

Due to the relation eq 18 (and eq [Disp-formula eqA.2]), the nonstationary flux evolution is key to understanding the time evolving population dynamics. Starting from a separable initial condition for the electronic and vibrational subspaces, ψ­(*t* = 0) = ψ_vib_({*x*_*n*_ = 0})|LE^*D*^⟩, a purely electronic flux *j* = *j*_*DA*_ rises rapidly during the first femtoseconds, i.e., on a time scale τ_el_ ∼ 2 fs (see Figure 4b and Figure S7 in the Supporting Information), before electronic-vibrational correlations start to build up. During this time scale τ_el_, the pure-state property remains conserved ( P=1 ), as can be inferred from Figure 4d.

Once electronic-vibrational correlations emerge, decoherence sets in, which we estimated from a short-time approximation to the purity decay^6,34,35^ at time *t* = τ_el_, i.e., P ­(*t* – τ_el_) = exp­[−(*t* – τ_el_)^2^τ_d_^–2^] with τ_d_ ∼ 32 fs, see Figure 4d and Figure S8 in the Supporting Information. Since we can assume that the system-bath state is still separable at time *t* = τ_el_, decoherence sets in quadratically from this time onward (see Sec. S3.3 in the Supporting Information). The Gaussian estimate is in good agreement with a short-time approximation for the spin-boson Hamiltonian,^34,35^ i.e., τ_d_^–2^ = 2⟨δ^2^ *Ŝ*⟩ $\left\langle \delta^{2}{Ê}_{\mathrm{DA}}\right\rangle$ = 2|*c*_*D*_|^2^|*c*_*A*_|^2^ *K*_Δ_^2^⟨*X̂*_Δ_^2^⟩, which represents the product of subsystem and bath variances taken with respect to the interaction Hamiltonian. The subsystem variance is given as ⟨δ^2^ *Ŝ*⟩ = |*c*_*D*_|^2^|*c*_*A*_|^2^, with *c*_*D*_ and *c*_*A*_ the electronic wavefunction coefficients at time *t* = τ_el_, and $\left\langle \delta^{2}{Ê}_{\mathrm{DA}}\right\rangle$ relates to the variance of energy gap fluctuations due to the bath modes.^34,35^ The latter can be expressed in terms of the variance of an effective mode, *K*_Δ_^2^⟨*X̂*_Δ_^2^⟩ = ∑_*s,n*_ κ_*n,s*_^2^⟨*x̂*_*n,s*_^2^⟩ (see Sec. S3.3 in the Supporting Information). This suggests a rapid decoherence process induced collectively by the vibrational modes, with a dominant participation of high-frequency modes. The decoherence time is slightly longer, though, than the value ∼10 fs estimated for the pure-dephasing case, with an initial 1:1 superposition state (see Sec. S3.3 and Figure S8 in the Supporting Information for details). The difference can mainly be attributed to the much smaller subsystem variance ⟨δ^2^ *Ŝ*⟩ in the case where the acceptor population just starts to emerge.

While the short-time estimate pertains to a Gaussian decoherence decay, the persistent interconversion of state populations and coherences due to the electronic coupling complicates the picture from ca. 10 fs onward. Decoherence is effectively slowed down due to the electronic coupling. Even so, at *t* ∼ 75 fs, where ρ_11_ = ρ_22_ = 0.5, the purity almost reaches the minimum of P=0.5 , corresponding to a fully decoherent state. At later times, the purity rises again as the population transfer is completed. Similar purity profiles were found in refs 36 and 37.

Around 200 fs, the flux and the real part of the electronic coherence reach a quasi-stationary state. At this point, around 80% of the *D* → *A* population transfer has happened. From this, we conclude that the EET transfer dynamics essentially falls into the time interval of coherent transients, as further explained below.

From 200 fs onward, a near-kinetic transfer regime sets in. The flux exhibits small oscillations around zero, whereas the real part of the coherence takes negative values, signaling a quasi-stationary coherent superposition of the |LE^*D*^⟩ and |LE^*A*^⟩ states. The transfer slows down and features a non-negligible temperature dependence, differently from the initial EET steps. With increasing temperature, the EET transfer decreases and the D/A superposition recedes, suggesting that a statistical mixed-state character is enhanced by temperature, slowing down nonadiabatic transfer. This is underscored by the purity whose asymptotic value decreases with temperature.

Taking into account the above observations, we carried out a three-time scale fit of the time-dependent population, beyond the initial electronic time scale τ_el_, as detailed in Sec. S3.1 in the Supporting Information. The relevant decay times are indicated in Figure 4. The initial electronic time scale, τ_el_ ∼ 2 fs is followed by a decoherence time scale, τ_d_ ∼ 32 fs, an ultrafast EET time scale, τ_1_^EET^ ∼ 105 fs, and a slower, kinetic EET time scale which depends on temperature, τ_2_^EET^ (*T*) ∼ 0.7–2.8 ps (see the intervals marked as “el”, “d”, “1”, and “2” in Figure 4). The coherent decay phase (τ_1_^EET^) is around an order of magnitude faster than the kinetic decay (τ_2_^EET^) and largely determines the net population transfer. While these time scales cannot be strictly separated, their combination accounts for the full population decay profile, as shown in Figure S6 in the Supporting Information.

To illustrate these time scales further, Figure 5 recasts the evolution of the electronic density matrix in terms of dynamics on the Bloch sphere, using the Pauli matrix representation of eq 19 and eq 20. The initial coherence created in the *xy*-plane on a time scale τ ∼ 25 fs decays rapidly such that the system evolves toward the *z*-axis. Importantly, the largest part of the EET transfer has already taken place when the flux (*y* component of the coherence, i.e., *j* = −γ_*DA*_ *s*_*y*_) has decayed around 200 fs. The subsequent kinetic-like time scale is marked by slow changes toward an asymptotic state. On the observation time scale, a small but non-negligible real (*s_x_*) component of the coherence is preserved asymptotically since a *D*/*A* superposition state is observed. However, the latter shrinks with increasing temperature (see Figure 4c and red traces in Figure 5).

**Figure 5:**
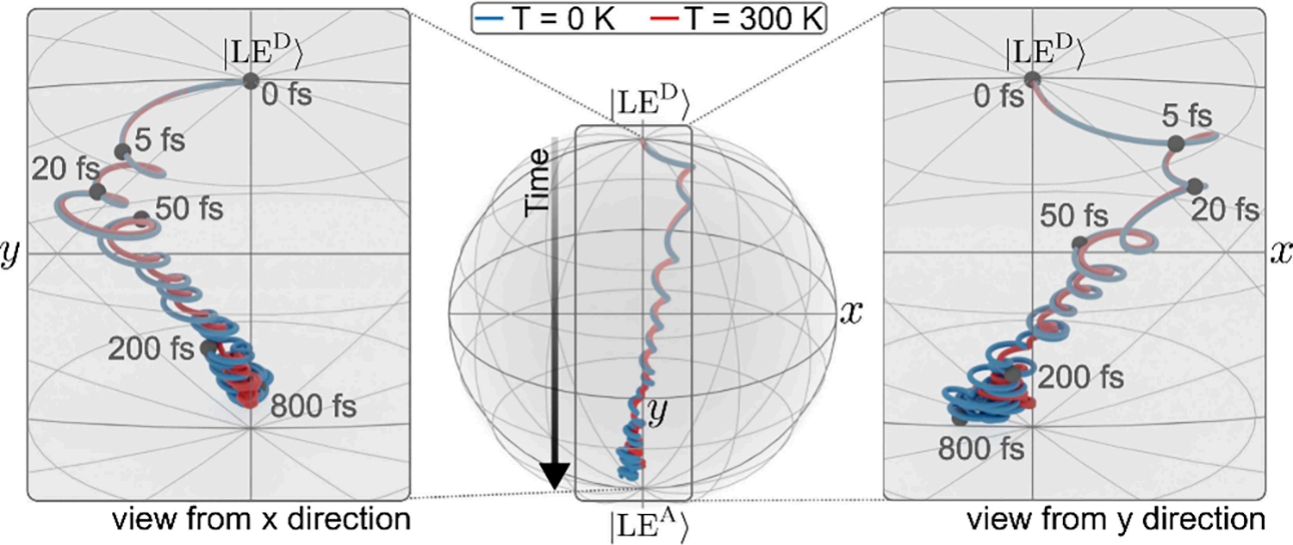
Representation of the EET dynamics on the Bloch sphere. At the center, the evolution from the donor population at the north pole to the acceptor population at the south pole is shown. On the l.h.s. and r.h.s., zoom-ins are shown where views are approximately taken from the *x*- and *y*-directions, illustrating the evolution of the *s*_*x*_ and *s*_*y*_ components of eq 20, which are related to the transient coherence and specifically to the electronic flux, *j* = −γ_*DA*_ *s*_*y*_.

## Mode Specificity and Vibronic Resonance

In order to better understand the mechanism at work on the fast EET time scale, τ_1_^EET^ ∼ 105 fs, we now analyze the manifestations of vibronic couplings. Since the donor vibrations turn out to play a dominant role, we focus on the latter in Figure 6, while the full set of vibrations is shown in Sec. S4.2 of the Supporting Information. Photoexcitation of the donor fragment initiates time-dependent displacements of the vibrations, whose amplitude depends on the vibronic coupling strength, as illustrated in panel a of Figure 6. Due to these displacements, crossings between the diabatic potential surfaces can be encountered, inducing nonadiabatic effects. However, the latter do not necessarily lead to an irreversible transfer between the donor and acceptor fragments. Typically, high-frequency vibrations (in the present case >1200 cm^–1^) induce sustained Rabi oscillations between potential surfaces exhibiting an energy offset.

**Figure 6:**
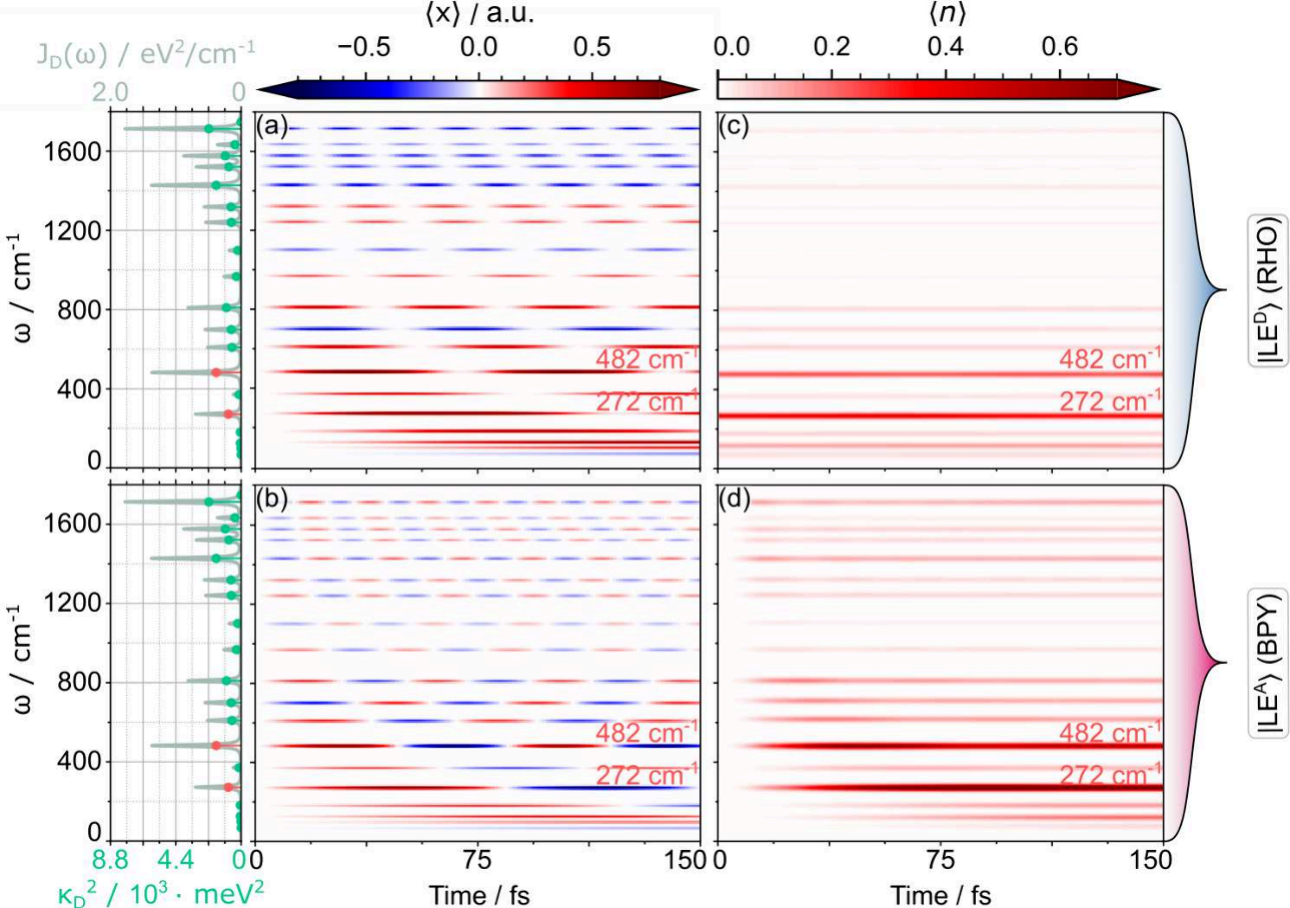
For the donor modes, state-specific displacements and mean occupation numbers are shown as a function of time; for reference, the donor SD, *J*_*D*_(ω), is shown on the l.h.s. a) Displacements in |LE_*D*_⟩ are pronounced for a number of modes in the donor state due to the photoexcitation, proportional to their vibronic couplings. b) These displacements persist in the |LE_*A*_⟩ state. c) Occupation numbers in the |LE_*D*_⟩ state and d) occupation numbers in the |LE_*A*_⟩ state. Occupation numbers are significant for modes with not-too-high frequencies and specifically for two modes (482 and 272 cm^–1^) where occupation numbers are close to 1 in the |LE_*A*_⟩ state. An analogous analysis for the acceptor modes is shown in Figures S10 and S11 in Sec. S4 in the Supporting Information.

Under specific circumstances, efficient mechanisms exist that can lead to the rapid dissipation of energy, inducing irreversible conversion |LE^*D*^⟩ → |LE^*A*^⟩. For weakly coupled aggregate systems, vibronic resonance effects^13,38–40^ can provide such a mechanism: here, a subset of vibrations are able to absorb a significant amount of energy, compensating for the energetic offset between high-frequency vibronic states. The presence of such modes is indicated by the time evolution of the occupation numbers, as shown in panels c and d of Figure 6. Indeed, several such modes exist in the present system, notably the donor modes that are labeled in the figure (ω_5,*D*_ = 272 cm^–1^, ω_7,*D*_ = 482 cm^–1^). These modes, which are illustrated in Figure 2, exhibit a pronounced athermal excitation with occupation numbers ⟨*n*⟩ ∼ 1. Combinations of these modes with high-frequency vibrations, notably (ω_5,*D*_ + ω_19,*D*_) and (ω_7,*D*_ + ω_16,*D*_), nearly match the electronic energy gap (see Table S1 in the Supporting Information for the full set of modes). Hence, the coherence evolution of Figure 4b-c is expected to feature multiphonon effects involving these vibronic frequencies, rather than the bare electronic frequency (ω_el_ ∼ |*ΔE*| = 0.25 eV = 2016 cm^–1^). These observations are supported by Fourier transformation of the coherence dynamics (see Sec. S5.1 in the Supporting Information), which reveals a number of frequency components that sustain a vibronic resonance effect, including the modes that were just mentioned.

Analysis of energy redistribution between the electronic and vibrational subspaces (see Sec. S3.4 in the Supporting Information) confirms that rapid electronic deexcitation with concomitant energy flow toward the vibrational subspace takes place within the first 100 fs. In the present model, mode-specific vibrational excitation persists due to the absence of other mechanisms of energy redistribution. That is, even after the EET process toward the acceptor is complete, the relevant donor modes remain in a vibrationally hot state as seen in Figure 6c-d.

## Minimal Vibronic Resonance Model

In order to illustrate the vibronic resonance mechanism, we make a further drastic simplification and reduce the LVC Hamiltonian to two effective modes (*x̂*_1,*D*_, *x̂*_2,*D*_), with *N*_*D*_ = 2 and *N*_*A*_ = 0, as described in detail in Sec. S6 in the Supporting Information. This model represents a short time approximation, and gives a qualitatively correct picture up to around 100 fs. As illustrated in Figure 7, the concerted evolution of a high-frequency effective donor mode (1493 cm^–1^) and a lower-frequency effective donor mode (544 cm^–1^) lead to an efficient, resonant energy transfer between donor and acceptor, noting that ω_1,*D*_ + ω_2,*D*_ ∼ |Δ*E*|. The lower-frequency mode ω_1,*D*_ is highly excited in this minimal model, around ⟨*n*⟩ ∼ 2.0, see Sec. S6.2 in the Supporting Information. This minimal model emphasizes that the transfer mechanism relies on (i) an initial encounter with the diabatic crossing seam, mainly driven by the high-frequency modes, followed by (ii) a nonadiabatic transition involving significant energy transfer to a lower-frequency mode. A very similar scenario has been discussed, e.g., in ref 13. While the present minimal 2-state model provides a useful illustration, a large number of modes are able to absorb energy in the full-dimensional system, leading to broader redistribution of vibrational energy. Even so, depending on the details of the structured SD, mode selectivity emerges even in a larger system, as illustrated in Figure 6c-d. Based on this observation, molecular fragments could be designed to feature absorbing modes in the desired frequency range. In the present case, the *D* fragment acts in a dual role, by initiating the EET event through large displacements of high-frequency modes, and acting as a vibrational energy acceptor in an intermediate frequency range. An acceptor fragment with a different spectral density could compete in the second role.

**Figure 7:**
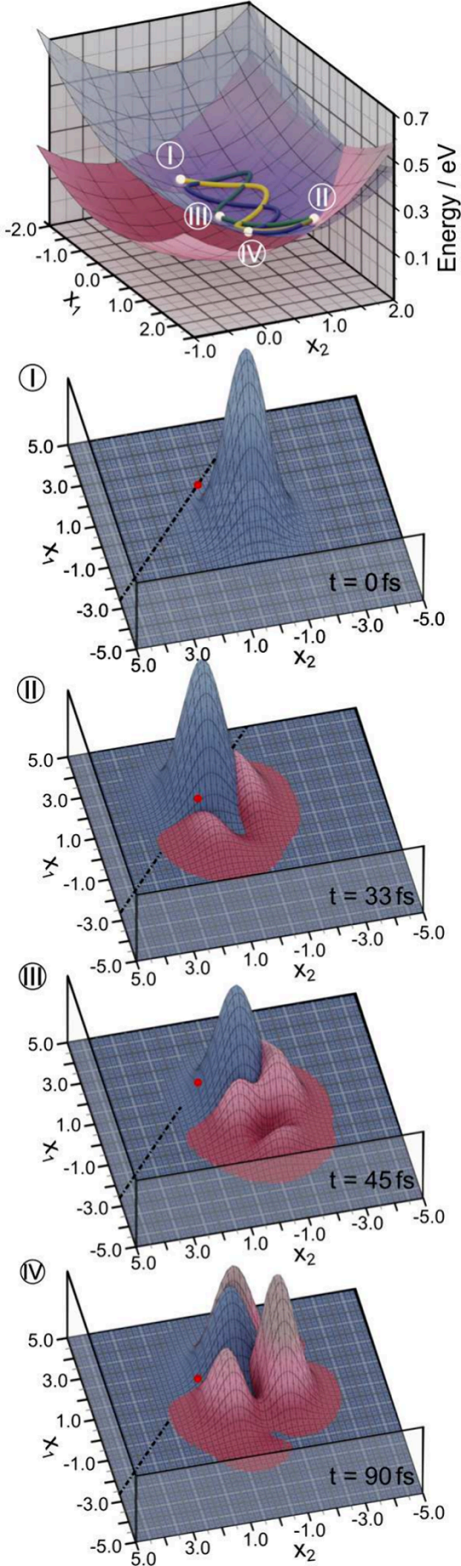
For the effective two-mode system, which is able to capture the short-time dynamics, time-evolving expectation values on the effective PESs (upper panel) along with snapshots of 2D densities (panels I to IV) are shown in the plane spanned by the two effective modes (*x*_1_, *x*_2_) = (*x*_1,*D*_, *x*_2,*D*_). (Note the difference in axis values and the flipped *x*_1_-axis in panels I to IV as compared with the uppermost panel.) Wavepacket density on the |LE_*D*_⟩ state is marked in blue, while density on the |LE_*A*_⟩ state is marked in magenta. The diabatic crossing seam is shown (dashed black line), along with the minimum energy point along the seam (red dot). It is seen that a highly vibrationally excited state is generated on the acceptor surface within tens of femtoseconds, with mean occupation number ⟨*n*⟩ ∼ 2.0 (see Figure S15 in Sec. S6.2 in the Supporting Information). This minimal two-mode model illustrates the vibronic resonance mechanism at work in the larger systems.

## Discussion

The present case study of a molecular dyad which has recently been investigated experimentally,^7^ illustrates how ultrafast and effective EET transfer across a comparatively large energy gap is mediated by an off-resonant vibrational environment provided by the molecular donor and acceptor fragments. It is seen that nonlinear processes, notably vibronic resonance effects, set in rapidly and lead to ultrafast decay dominated by a subset of active vibrational modes. The latter remain in an athermal hot state after the EET transfer has occurred. These modes would gradually cool off in a more complete model including anharmonicity effects and interaction with a solvent environment. Following this initial transfer phase on a time scale of ∼200 fs where around 80% of the donor-to-acceptor transfer occurs, a quasi-stationary phase of the dynamics with significantly slower transfer sets in. While we do not expect a significant influence of a solvent environment on the ultrafast EET conversion and decoherence processes,^30^ besides shifts in the energy gap,^7^ the longer time scales are likely influenced by a solvent.

From the vantage point of system-bath theory, the present dynamics of a spin-boson type system is highly non-Markovian, since the EET dynamics occurs on the time scale of the initial transients. This is best illustrated by the evolution on the Bloch sphere depicted in Figure 5, where a persistent flux accompanies the system’s evolution from the north pole to the south pole. The flux evolution, in turn, is strongly dependent on the initial coherence generated by purely electronic evolution during <5 fs, along with the initial conditions of the vibrational subsystem, which features large photoinduced displacements of a subset of vibrations. Due to the vibronic resonance processes occurring during this initial time, the transfer dynamics is almost completed before the transients have died down. In this sense, the EET process in the present system is intermediate between a coherent and kinetic regime. Also, due to the lack of a separation of time scales, the flux evolution does not suggest the identification of a plateau value which would define a transfer rate for the EET process.

In recent work,^41^ combined experimental and theoretical studies have been carried out on related donor–acceptor dyads with different molecular composition. In these systems, EET was found to be significantly slower, between 1 and 3 ps or more. These systems feature a larger electronic energy gap (|*ΔE*| ∼ 0.33 eV) and smaller electronic couplings (γ_*DA*_ ∼ 0.005–0.008 eV), presumably rendering ultrafast vibronic resonance effects less favorable. In line with this observation, the flux evolution in these systems does not show a two-time scale character as in the present system.^41^ If ultrafast and efficient EET is desired, energy gap engineering could be combined with the tailoring of molecular spectral densities in order to optimize the relevant donor–acceptor architectures.

Finally, it is worth pointing out that large photoexcited molecular systems exhibiting a dense manifold of excited states likely exhibit transitions between an initial coherent decay phase, showing a marked quantum flux, and slower, decoherent decay at longer times.^42^ In this context, phonon bottlenecks may occur, due to a mismatch between electronic energy gaps and vibrational energies. These can be bridged by nonlinear (multiphonon) effects^43^ similar to those observed in the present system.

## Supplementary Material



## Appendix

### Transient State-to-State Flux

The definition of the transient state-to-state flux of eq 17 can be straightforwardly obtained from the Liouville-von-Neumann (LvN) equation for the density operator, *ρ̂*(*t*) = |*Ψ*(*t*)⟩⟨*Ψ*(*t*)|, where *Ψ*(*t*) is the 2L-GMCTDH wavefunction specified in eq 13

A.1
$$\frac{\partial \mathrm{\rho ̂}}{\partial t}=-i\left[{Ĥ}^{\mathrm{LVC}},\mathrm{\rho ̂}\left(t\right)\right]$$

We now consider the electronic density matrix defined in eq 15, i.e., *ρ̂*^el^ = Tr_vib_ *ρ̂*, whose diagonal elements *ρ*_*ss*_^el^ represent state populations while the off-diagonal elements *ρ*_*ss*′_^el^, *s* ≠ *s*′, represent coherences. From the LvN equation, we find for the temporal change of the electronic state population of the *s*th state

A.2
dρsseldt=−iTrvib⟨s|[ĤLVC,ρ̂(t)]|s⟩=−iTrvib∑s′≠sγss′⟨s|[(|s⟩⟨s′|+|s′⟩⟨s|),ρ̂(t)]|s⟩=2∑s′γss′ImTrvib{ρs′s(t)}=2∑s′γss′Im⁡ρs′sel(t)≡∑s′js′s(t)

In the above expression, only the coupling terms of the LVC Hamiltonian are found to contribute on the r.h.s., which are here formulated for a general *N*-state electronic Hamiltonian *Ĥ* ^el^ = ∑_*s*_ *ΔE*_*s*_|*s*⟩⟨*s*| + ∑_*ss*′_ *γ*_*ss*′_(|*s*⟩⟨*s*′| + |*s*′⟩⟨*s*|), which reduces to the electronic two-state Hamiltonian of eq 2 in our case. From these expressions, the state-to-state flux *j*_*s*′*s*_(*t*) is identified in terms of the coherence elements, i.e., *j*_*s*′*s*_(*t*) = 2*γ*_*ss*′_ Im *ρ*_*s*′*s*_^el^(*t*), *s* ≠ *s*′. This expression only requires the electronic coupling part of the Hamiltonian, i.e., the effect of the bath on the state-to-state flux only resides in its effect on the coherence at time *t*, *ρ*_*s*′*s*_^el^(*t*). Note that, from the second line of eq [Disp-formula eqA.2] onwards, it has been assumed that the coupling is coordinate-independent; a more general expression is given in ref 42, where coordinate-dependent coupling terms were considered. Furthermore, one should point out that the above relation for the flux holds for time reversal invariant Hamiltonians, as is the case for standard vibronic coupling Hamiltonians with real-valued state-to-state coupling terms (see the discussion in Sec. S7 in the Supporting Information).

## Notes

Details of the reference spectral density, effective-mode transformation scheme, and convergence properties; the full set of effective modes employed in the model; 2L-GMCTDH calculation details; details of the multiple-time scale EET dynamics; displacement maps and occupation numbers for the full set of modes; vibronic resonance effects and mode specificity details; time evolution of the minimal two-effective mode system; and a more general formulation of the state-to-state flux (PDF)
