# Supplementary material for: Excitation Energy Transfer in an Intermediate Regime: A Multiconfigurational Gaussian Wavepacket Study of a Light-Harvesting Supramolecular Dyad
Source: J Phys Chem Lett. 2026 Mar 9;17(11):3117–26. doi: 10.1021/acs.jpclett.6c00100 (PMC13007025; doi:10.1021/acs.jpclett.6c00100)
Supplement: Supplementary file 1 [file jz6c00100_si_001.pdf]

Supporting Information: Excitation Energy Transfer in an Intermediate Regime: A Multiconfigurational Gaussian Wavepacket Study of a Light-Harvesting Supramolecular Dyad

Sreeja Loho Choudhury,[†,¶] Maximiliane Horz,[†,¶] Rainer Hegger,[†] Rocco Martinazzo,[‡] and Irene Burghardt[*,†]

†*Institute of Physical and Theoretical Chemistry, Goethe University Frankfurt, Max-von-Laue-Str. 7, 60438 Frankfurt, Germany*

‡*Department of Chemistry, Università degli Studi di Milano, Via Golgi 19, 20133 Milano, Italy*

¶These authors contributed equally.

E-mail: burghardt@chemie.uni-frankfurt.de

Contents

S1 Effective mode representation **S2**
S1.1 Spectral density of RHO-BPY supramolecular dyad S2
S1.2 Effective modes and spectral densities S4
S1.3 40-effective-mode representation S4

S2 Multiconfigurational 2L-GMCTDH calculations **S8**
S2.1 2L-GMCTDH tree representation S8
S2.2 Comparison 2L-GMCTDH vs. MCTDH S11

S3 Details of EET dynamics **S13**
S3.1 Time scales of population transfer S13
S3.2 Initial electronic time scale S15
S3.3 Decoherence and purity decay S15
S3.4 Energy redistribution S18

S4 Vibrational displacements and occupation numbers **S18**
S4.1 State-specific mode displacements S18
S4.2 State-specific occupation numbers S19

S5 Mode specificity and vibronic resonance effects **S21**
S5.1 Fourier analysis of coherence evolution S21
S5.2 Exclusion of subsets of modes from the dynamics S22

S6 Reduced two-effective mode dynamics **S23**
S6.1 Population decay, coherence, purity S24
S6.2 Mode displacements and occupation numbers S24
S6.3 2D densities in comparison with MCTDH S26

S7 State-to-state flux **S26**

References **S27**

S1 Effective mode representation

S1.1 Spectral density of RHO-BPY supramolecular dyad

As explained in the main text, a linear vibronic coupling (LVC) model was parametrized in Ref. 1 (see the Supporting Information of this reference), based on the full set of normal modes of the supramolecular dyad system. Linear vibronic coupling constants are computed from Franck-Condon gradients, in the full dimensionality of the normal-mode space.[1] Due to the rigid alkyne bridge between the donor (D) and acceptor (A) fragments, the normal modes separate into two subsets with 149 donor modes and 117 acceptor modes, respectively, such that the vibronic Hamiltonian reads as follows,[1]

$$\hat{H}^{\text{el-vib}} \;=\; \sum_{n=1}^{149} \kappa_{n,D}^{(0)} \hat{x}_{n,D} |\text{LE}^D\rangle\langle\text{LE}^D| + \sum_{n=1}^{117} \kappa_{n,A}^{(0)} \hat{x}_{n,A} |\text{LE}^A\rangle\langle\text{LE}^A| \tag{S1}$$

[1]For a total number of 91 atoms of the donor-acceptor dyad, the number of vibrational normal modes is 267. However, a torsional mode on the RHO fragment was discarded since it was associated with an imaginary frequency using the long-range corrected CAM-B3LYP functional that was chosen for the excited-state gradient computation, see the discussion in Ref. [1]. As a result, the total number of vibrational modes that was used in the quantum dynamical calculations was 266 rather than 267 in Ref. [1].

Mass- and frequency weighted coordinates are used throughout, such that the coordinates are dimensionless.

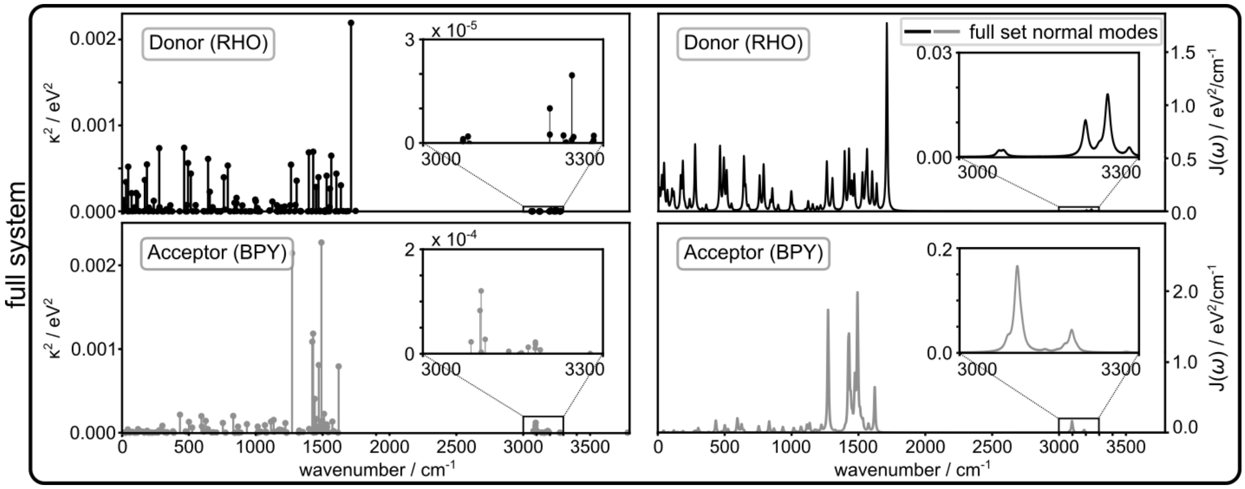

Figure S1: Discrete spectral densities (l.h.s.) along with continuous versions (r.h.s.) of the same SDs obtained by convolution with a Lorentzian function, are shown for the original set of normal modes with 149 donor modes and 117 acceptor modes. Note the spectral gap between ∼1750 cm^{-1} and ∼3050 cm^{-1}, where the high-frequency modes above 3050 cm^{-1} correspond to C-H and O-H modes.

In Figure S1 (l.h.s. panels), the discrete vibronic couplings are shown for the full-dimensional system,

$$J_D(\omega) = \frac{\pi}{2}\sum_{n=1}^{149}(\kappa_{n,D}^{(0)})^2\,\delta(\omega-\omega_{n,D}) \qquad ; \qquad J_A(\omega) = \frac{\pi}{2}\sum_{n=1}^{117}(\kappa_{n,A}^{(0)})^2\delta(\omega-\omega_{n,A}) \tag{S2}$$

Continuous versions of these SDs are shown on the r.h.s., obtained by convolution with a Lorentzian with full width at half maximum (FWHM) of 5 cm^{-1}.

The spectral densities exhibit a gap between ∼1750 cm^{-1} and ∼3050 cm^{-1}, since there are high-frequency modes that appear above 3050 cm^{-1} (0.38 eV), corresponding to C-H and O-H modes. Specifically, the RHO moiety displays 21 modes in this window while the BPY moiety displays 18 modes, leading to 39 modes overall in this frequency range. These modes were included in our time-dependent simulations of Ref. [7], but they were found to play a negligible role in the EET dynamics, due to their small vibronic couplings and their lack of resonance with the electronic energy gap. For this reason, the relevant modes were disregarded in the subsequent effective-mode analysis (see following subsection), along with several weakly coupled low-frequency modes (i.e., 6 modes on the BPY moiety and 7 modes on the RHO moiety below 50 cm^{-1}).

As a result, the reference donor and acceptor spectral densities (SDs) for the present study contain $N_D^{(0)} = 121$ donor modes and $N_A^{(0)} = 93$ acceptor modes, respectively.

S1.2 Effective modes and spectral densities

Following Refs. [2–4], a systematic reduction in dimensionality of the SDs can be achieved by effective-mode transformations. In the case of the donor-acceptor dyad system, where separate subsets of modes are coupled to the electronic states, separate effective-mode transformations are carried out in these subspaces. That is, the vibronic interaction is transformed to collective variables $\hat{X}_1^D$ and $\hat{X}_1^A$ which subsume all vibronic couplings,

$$\begin{aligned}\hat{H}^{\text{el-vib}} &= \sum_{n=1}^{N_D^{(0)}} \kappa_{n,D}^{(0)} \hat{x}_{n,D} |\text{LE}^D\rangle\langle\text{LE}^D| + \sum_{n=1}^{N_A^{(0)}} \kappa_{n,A}^{(0)} \hat{x}_{n,A} |\text{LE}^A\rangle\langle\text{LE}^A| \\ &\equiv K_1^D \hat{X}_1^D |\text{LE}^D\rangle\langle\text{LE}^D| + K_1^A \hat{X}_1^A |\text{LE}^A\rangle\langle\text{LE}^A| \end{aligned} \quad \text{(S3)}$$

where $\hat{X}_1^D = (K_1^D)^{-1} \sum_{n=1}^{N_D^{(0)}} \kappa_{n,D}^{(0)} \hat{x}_{n,D}$, with $K_1^D = \sqrt{\sum_n (\kappa_{n,D}^{(0)})^2}$, and analogously for $\hat{X}_1^A$ and K_1^A.

As mentioned above, the high-frequency modes > 3050 cm^{-1}, along with several low-frequency modes < 50 cm^{-1}, were excluded from the construction of reduced-dimensional SDs since these modes were found to have a negligible influence on the dynamics in our earlier study.[1] Hence, $N_D^{(0)} = 121$ and $N_A^{(0)} = 93$ in Eq. (S3) as explained in the preceding subsection.

The collective variables $\hat{X}_1^D$ and $\hat{X}_1^A$ are in turn, coupled to a remaining set of residual modes $\hat{X}_n^D$, $n = 2, \dots, N_D^{(0)}$, and $\hat{X}_n^A$, $n = 2, \dots, N_A^{(0)}$. The latter can be represented in a hierarchical chain representation with bilinear couplings. If the chain is truncated at a given order and the Hessian matrix in the effective-mode space is re-diagonalized,[4] a reduced-dimensional LVC Hamiltonian in standard representation is obtained, where all modes are again coupled to the electronic subsystem,

$$\hat{H}_{\text{eff}}^{\text{el-vib}} = \sum_{n=1}^{N_D} \kappa_{n,D} \hat{x}_{n,D} |\text{LE}^D\rangle\langle\text{LE}^D| + \sum_{n=1}^{N_A} \kappa_{n,A} \hat{x}_{n,A} |\text{LE}^A\rangle\langle\text{LE}^A| \quad \text{(S4)}$$

The above procedure is employed to generate reduced-dimensional versions of the SDs $J_D(\omega)$ and $J_A(\omega)$. In Fig. S2 (middle and lower panels), reduced-dimensional SDs for $N_D = N_A = 40$ modes and $N_D = N_A = 20$ modes are illustrated. In the implementation of the present paper, the latter case of 40 modes, with $N_D = N_A = 20$, is considered.

S1.3 40-effective-mode representation

Table S1 lists the mode frequencies and vibronic couplings appearing in the reduced-dimensional linear vibronic coupling Hamiltonian Eq. (S4) for $N_D = N_A = 20$, i.e., 40 effective modes overall. The l.h.s. part of the table contains the modes coupled to the acceptor state, while the r.h.s. part contains modes coupled to the donor state, respectively. In Figure

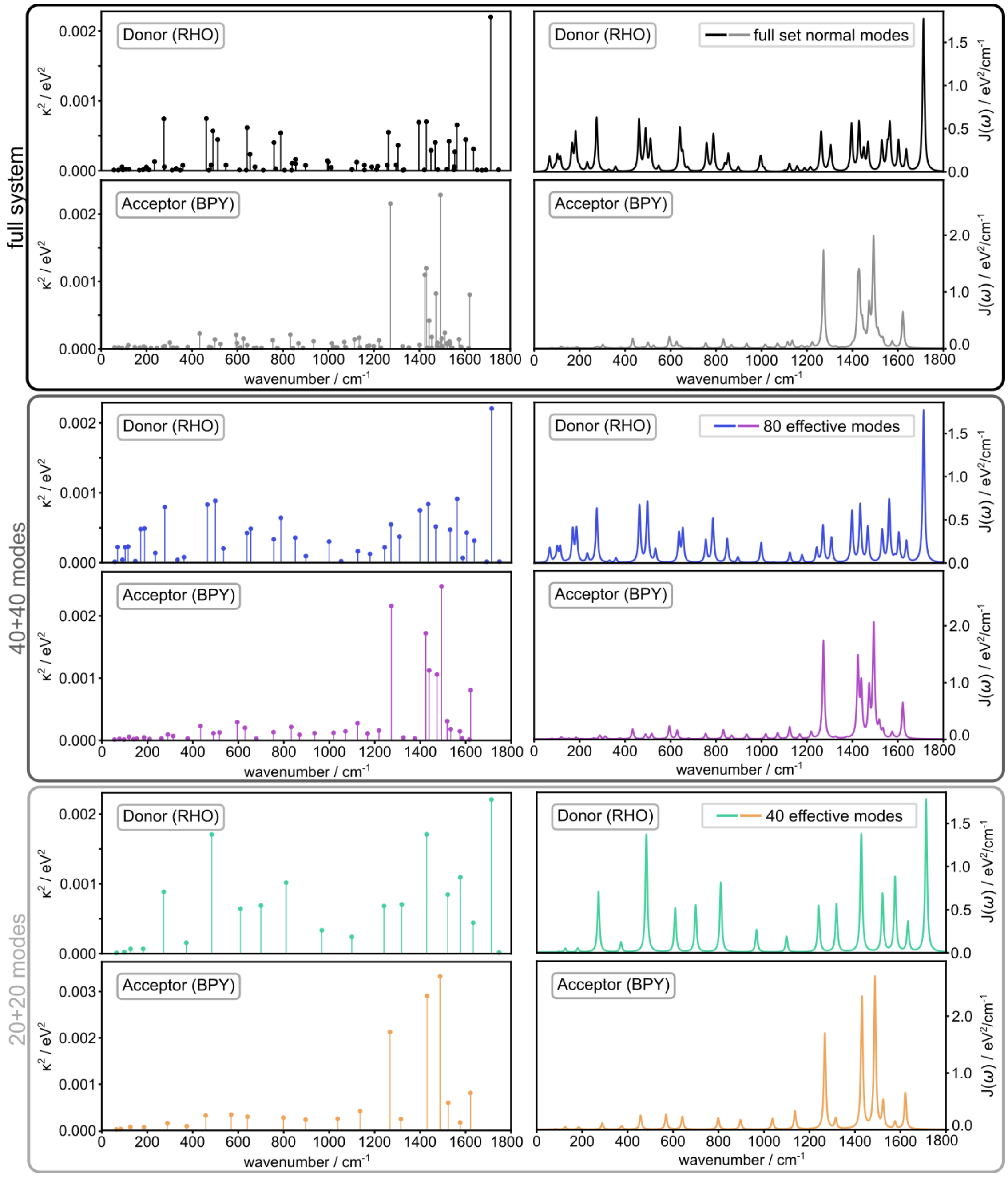

Figure S2: Discrete spectral densities (l.h.s.) along with continuous versions (r.h.s.) of the same SDs obtained by convolution with a Lorentzian function, are shown for the original set of normal modes with $N_D^{(0)} = 121$ and $N_A^{(0)} = 93$ within the relevant frequency window (upper panels), a reduced-dimensional version with $N_D = 40$ and $N_A = 40$ (middle panels), and the version employed in our analysis, $N_D = 20$ and $N_A = 20$ (lower panels).

S3, these modes are illustrated in an atomistic representation in terms of their Cartesian displacements.

In the finite-temperature calculations reported in the main text, four modes of the D and A fragments, respectively, are thermalized; the frequencies of these modes lie below $k_B T$ at room temperature. Within the thermofield dynamics (TFD) approach, auxiliary "tilde" modes are included such that the total number of modes in the thermalized calculations amounts to 48 modes overall. The full TFD-based LVC Hamiltonian is indicated in Eqs. (10)-(12) of the main text.

Table S1: Frequencies and vibronic couplings of the effective modes for the reduced-dimensional $N_D = N_A = 20$ system. The relevant Hamiltonian is given in Eq. (S4). The four l.h.s. columns refer to the modes coupled to the acceptor (A) fragment, while the four r.h.s. columns refer to the modes coupled to the donor (D) fragment. The first four modes of the D and A fragments, separated by a horizontal line, are those thermalized by the TFD approach, such that 48 modes appear overall in the thermal calculations.

mode	ω [meV]	ω [cm^{-1}]	κ [meV]	mode	ω [meV]	ω [cm^{-1}]	κ [meV]
M_1^A	7.9	64	−1.7	M_1^D	8.3	67	−1.4
M_2^A	10.3	83	3.6	M_2^D	12.2	98	3.2
M_3^A	15.5	125	7.4	M_3^D	15.5	125	7.4
M_4^A	23.0	186	−7.1	M_4^D	22.4	181	7.5
M_5^A	35.7	285	−11.7	M_5^D	33.7	272	29.6
M_6^A	46.3	373	8.6	M_6^D	46.0	371	12.0
M_7^A	56.7	457	−17.5	M_7^D	59.8	482	41.2
M_8^A	70.5	569	18.0	M_8^D	75.5	609	25.2
M_9^A	79.4	640	−16.7	M_9^D	86.6	698	−26.0
M_{10}^A	99.0	798	16.0	M_{10}^D	100.4	810	31.7
M_{11}^A	111.1	896	−14.6	M_{11}^D	119.9	967	18.0
M_{12}^A	128.6	1037	−15.3	M_{12}^D	136.2	1099	−15.1
M_{13}^A	140.9	1136	20.0	M_{13}^D	153.8	1240	25.9
M_{14}^A	157.2	1268	45.9	M_{14}^D	163.5	1387	26.4
M_{15}^A	163.1	1315	15.2	M_{15}^D	177.0	1428	−41.2
M_{16}^A	177.4	1431	−53.8	M_{16}^D	188.6	1521	−28.9
M_{17}^A	184.5	1488	−57.5	M_{17}^D	195.5	1577	−32.9
M_{18}^A	188.9	1524	24.1	M_{18}^D	202.4	1632	−20.8
M_{19}^A	195.4	1576	12.3	M_{19}^D	212.4	1713	−46.9
M_{20}^A	201.1	1622	28.2	M_{20}^D	216.6	1747	2.2

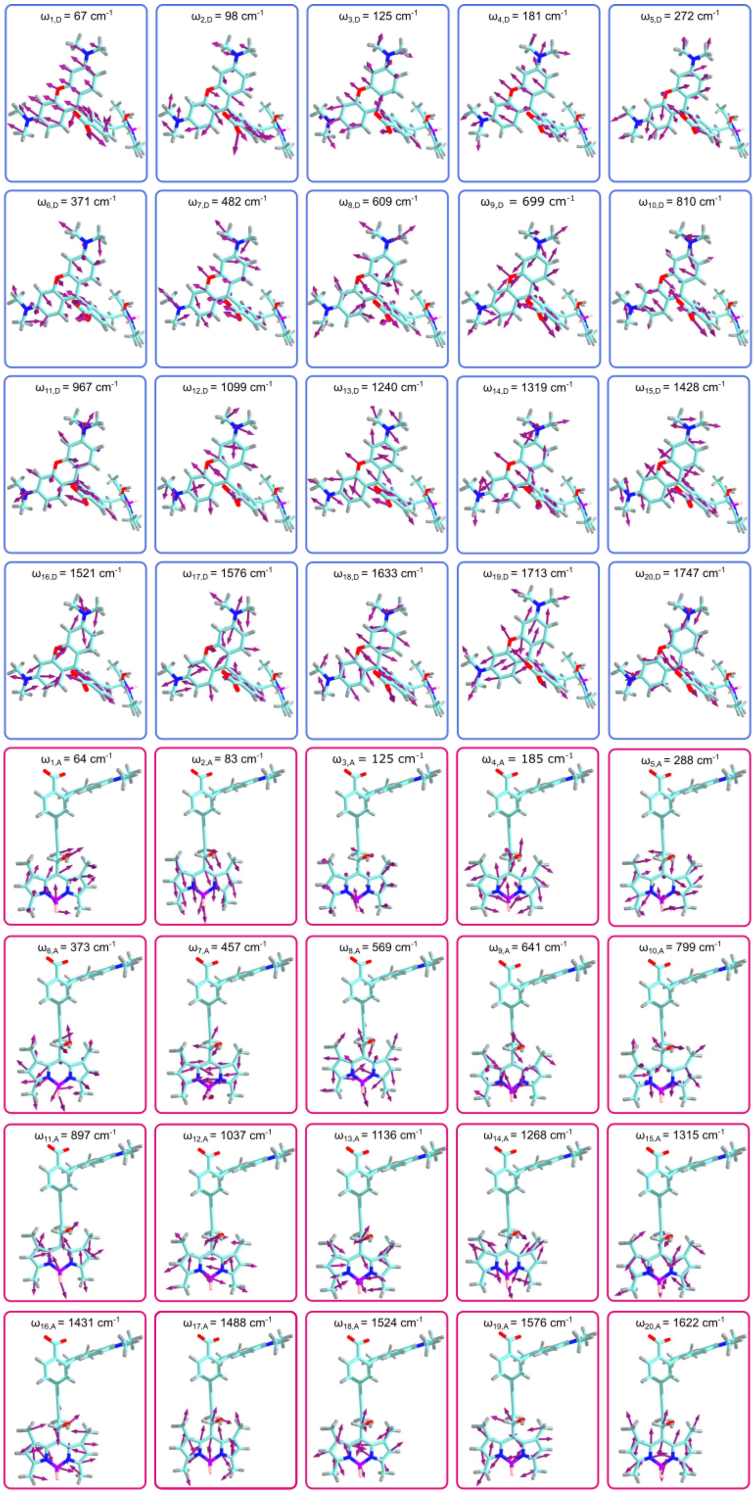

Figure S3: Representation of characteristic effective mode motions in terms of mass-weighted Cartesian displacements. The first 20 modes (with blue frames) represent the donor modes, while the second 20 modes (with red frames) the acceptor modes, respectively.

S2 Multiconfigurational 2L-GMCTDH calculations

S2.1 2L-GMCTDH tree representation

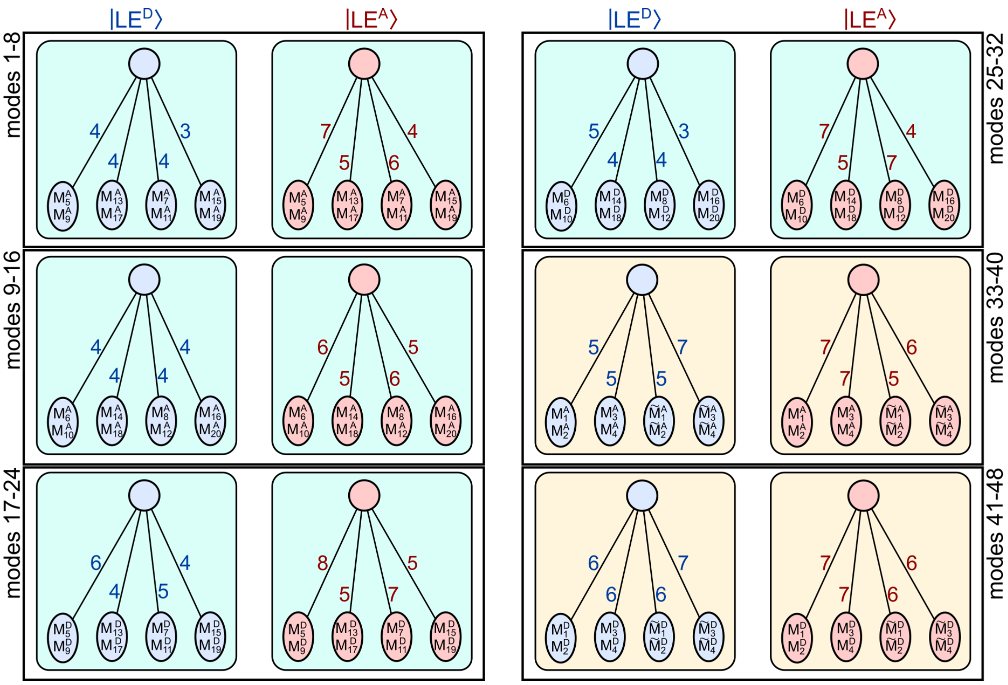

Figure S4: Complementary to the representation of the 2L-GMCTDH tree given in Figure 3 of the main text, details are given for the decomposition of first-layer modes into second-layer GWP particles. Each second-layer GWP consists of two modes, which are either of real or tilde type. That is, 20 GWPs represent 40 real modes while 4 GWPs represent 8 auxiliary "tilde" modes. The modes $\mathrm{M}_i^{A/D}$ indicated in the ellipses are assigned according to Table S1, while the additional modes $\tilde{\mathrm{M}}_i^{A/D}$ denote the auxiliary $\tilde{M}_n^A$ particles, respectively. As in the main text, the acceptor state number is given in red, while the donor state one is given in blue. Numbers placed along the lines correspond to the number of second-layer GPWs that are part of the first-layer particle of the same coloring.

Here we provide details on the computational set-up. Complementary to the 2L-GMCTDH tree of Figure 3 in the main text, Fig. S4 illustrates the decomposition of the first-layer particles into second-layer GWPs. It should be mentioned that the same set-up – including tilde modes – is used for all temperatures, including $T = 0K$ where the tilde modes are uncoupled from the electronic subsystem. We verified that no numerical instabilities result in this case. The main advantage of this approach is that the model-size dependent errors are the same for all temperatures. The numbers of first-layer and second-layer modes are adapted to the highest-temperature calculations which carry the strongest correlations. The computational details shown in Table S2 apply to the calculations at all temperatures.

To quantify the convergence of the 2L-GMCTDH calculations we monitor the natural orbital populations of the first-layer– and the second-layer–modes. Due to the construction of

Table S2: Computational details of the simulations shown in the main text. Since the same tree structure and numbers of first and second-layer particles are used for 48-mode calculations (i.e., 40 real modes plus 8 tilde modes) at all temperatures, the data are the same for all T values.

CPU time	302 hours
main memory	2 GB
number of A coefficients	3122400
number of B coefficients	115701
number of GWPs	512
disk space A coefficients	125 GB
disk space B coefficients	4.4 GB
disk space GWPs	59 MB

the 2L-GMCTDH method, the first layer single particle functions (SPFs) are orthonormal. Thus the natural orbital populations are directly obtained from reduced density matrices based on the corresponding single–particle modes. The second-layer particles of GWP type are non-orthogonal such that a Löwdin transformation[5] of the reduced density matrix of each (κ,μ)–subspace[6,7] was performed to orthonormalize the basis, i.e.,

$$\boldsymbol{\rho}_{\text{orth}}^{(\kappa,\mu)} = \boldsymbol{S}^{1/2}\boldsymbol{\rho}^{(\kappa,\mu)}\boldsymbol{S}^{1/2}$$

where $\boldsymbol{S}$ is the overlap matrix of the GWPs within the (κ,μ)–subspace and $\boldsymbol{\rho}^{(\kappa,\mu)}$ is the corresponding density matrix. The eigenvalues of $\boldsymbol{\rho}_{\text{orth}}^{(\kappa,\mu)}$ then correspond to the natural orbital populations.

The results are shown in Table S3 for the first layer (L1) particles and in Table S4 for the second layer (L2) particles, for all temperatures. The tables contain the highest natural population of the simulation as column 4 while column 2 shows on which electronic state this maximum population appeared and column 3 notes which particle shows this maximal value. In case of Table S3 the particle is specified by the number of the L1 particle while in Table S4 column 2 shows a L1/L2 combination. The last column in both tables shows the number of particles whose natural population exceeds a given threshold. This threshold is set to $2 \cdot 10^{-3}$ ($|\mathrm{LE}^A\rangle$) and $1 \cdot 10^{-3}$ ($|\mathrm{LE}^D\rangle$), respectively, for the L1 particles and to 10^{-4} for the L2 particles.

Table S3: The table shows the first layer particle (SPF) with the highest natural orbital population for all temperatures in both donor and acceptor states. Column 1 indicates the temperature (T); in the following columns, the l.h.s. part (columns 2-4) refer to the $|\mathrm{LE}^A\rangle$ state, while the r.h.s. part (columns 5-7) refers to the $|\mathrm{LE}^D\rangle$ state. Column 2/5: number of first layer particle that shows the maximal natural orbital population, and column 3/6: natural orbital population of this particle. Column 4/7: Number of first layer particles for which the natural orbital population is larger than $2 \cdot 10^{-3}$ and 10^{-3}, respectively.

	$\|\mathrm{LE}^A\rangle$			$\|\mathrm{LE}^D\rangle$		
T [K]	L1 particle	max. natpop	# natpop $> 2 \cdot 10^{-3}$	L1 particle	max. natpop	# natpop $> 10^{-3}$
0	5	$3.2 \cdot 10^{-3}$	2	3	$1.1 \cdot 10^{-3}$	1
100	5	$3.9 \cdot 10^{-3}$	2	3	$1.1 \cdot 10^{-3}$	2
200	5	$3.0 \cdot 10^{-3}$	2	1	$2.0 \cdot 10^{-3}$	3
300	5	$3.1 \cdot 10^{-3}$	4	1	$2.7 \cdot 10^{-3}$	2

Table S4: Analogously to Table S3, the natural orbital populations are shown for the second layer (L2) GWP particles. Columns 2/5 now refer to a particle pertaining to L2 within an L1 particle. The threshold for the last column is set to 10^{-4} for these particles.

	$\|\mathrm{LE}^A\rangle$			$\|\mathrm{LE}^D\rangle$		
T [K]	L1/L2 particle	max. natpop	# natpop $> 10^{-4}$	L1/L2 particle	max. natpop	#natpop $> 10^{-4}$
0	4/3	$2.1 \cdot 10^{-4}$	3	5/3	$2.1 \cdot 10^{-4}$	4
100	3/2	$1.6 \cdot 10^{-4}$	4	1/2	$2.9 \cdot 10^{-4}$	6
200	1/3	$2.9 \cdot 10^{-4}$	4	3/1	$2.1 \cdot 10^{-4}$	7
300	1/2	$1.2 \cdot 10^{-3}$	5	1/2	$4.6 \cdot 10^{-4}$	6

The problem of linear dependencies of the non-orthogonal "frozen" GWPs was counteracted by modifying the width parameter, i.e., the constant a_{ni} parameter in the *ansatz* $g_{ni}(x_n, \Lambda_{ni}(t)) = \exp(a_{ni} x_n^2 - \xi_{ni}(t) x_n + \eta_{ni}(t))$ for the ith GWP representing the nth degree of freedom. This regularization scheme has already been applied in the first applications of 2L-GMCTDH in Refs. [6,7]. Two GWPs become linearly dependent if they strongly overlap since their parameters a_{ni} and $\xi_{ni}(t)$ become very similar. As a result, the overlap matrix of the second layer SPFs becomes ill conditioned. The values of the complex parameter $\xi_{ni}(t)$ which encodes coordinates and momenta are determined by the equations of motion and are thus not adjustable. However, the overlap can be reduced by varying the a_{ni} values. To this end, we chose the following procedure: Given that the ground-state width for a harmonic mode in mass-frequency weighted coordinates is $a_{ni} = -0.5$, we assign this value to the first GWP ($i = 1$ for mode x_n). For additional GWPs, a_{ni}, $i > 1$, is multiplied by an increasing power of a factor f or it is divided by an increasing power of that factor. The larger the factor f, the smaller the GWP overlap becomes. But if f is too large, the a_{ni} width parameters get quickly either very small or very large, which make them less relevant for the wave-function. Thus one needs to find a compromise that works best for the problem at hand. In our case we chose $f = 1.25$, with a series of a parameters shown in Table S5, obtained by successive multiplication and/or division by f.

Table S5: Width parameters for GWPs employed in the subspaces defined by the 2L-GMCTDH method.

$a_{1,n}$	$a_{2,n}$	$a_{3,n}$	$a_{4,n}$	$a_{5,n}$	$a_{6,n}$	$a_{7,n}$
-0.5	-0.625	-0.4	-0.32	-0.78	-0.98	-0.26

S2.2 Comparison 2L-GMCTDH vs. MCTDH

The multi-set variant of the 2L-GMCTDH approach is closely related to multi-set MCTDH, where state-specific basis sets are employed. In contrast, single-set MCTDH and single-set multi-layer ML-MCTDH employ state-independent single-particle functions. Therefore, we choose to carry out a comparison between multi-set 2L-GMCTDH and multi-set MCTDH calculations.

In order to verify the results obtained with multi-set 2L-GMCTDH by comparison with multi-set MCTDH, we constructed a lower-dimensional variant of our system, comprising 18 effective modes (9 modes coupled to the $|\mathrm{LE}^D\rangle$ and $|\mathrm{LE}^A\rangle$ states, respectively). The four lowest-frequency modes, with frequencies below $k_B T$ at 300 K, are thermalized. This model exhibits a very similar dynamics as the 40-mode model addressed in the main text.

The results of the dynamical calculations are shown in Fig. S5. The quality of the simulations in terms of the natural orbital populations are comparable. The largest natural orbital population was of the order of 10^{-3} for both methods. One clearly sees that the multi-set 2L-GMCTDH and the multi-set MCTDH are in good agreement, even though deviations

appear at finite temperature, especially regarding the real part of the electronic coherence which is reduced more rapidly towards longer times in the case of 2L-GMCTDH. The reduction of $\text{Re}\rho_{DA}(t)$ can be attributed to the thermal destruction of coherent superposition properties.

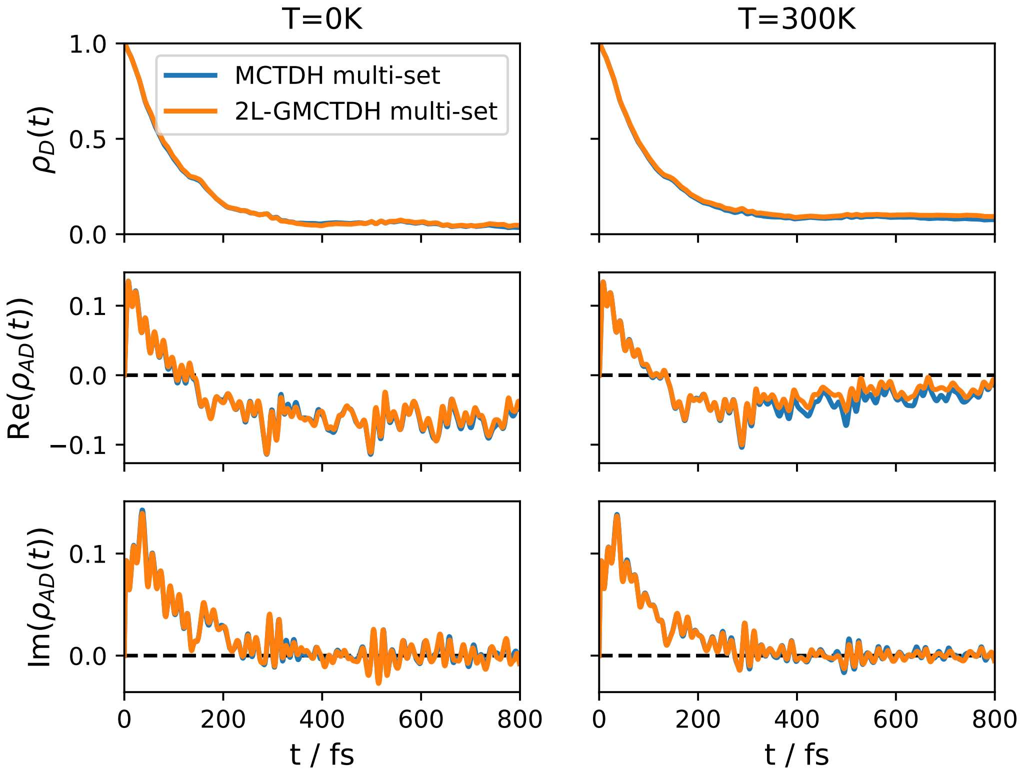

Figure S5: For $T = 0$ K and $T = 300$ K, the $|\text{LE}^D\rangle$ state population and real and imaginary parts of the electronic coherence are compared for multi-set 2L-GMCTDH and multi-set MCTDH calculations, for the 18+4-mode model as discussed in the text.

We also conducted comparisons with the ML-MCTDH approach,[8] which is usually employed in a single-set framework. Here, we found larger differences with respect to the multi-set 2L-GMCTDH calculations, especially regarding the real part of the electronic coherence. In the single-set ML-MCTDH approach, $\text{Re}\rho_{DA}(t)$ does not reduce significantly at finite temperature. The observed difference most likely relates to differences in the convergence properties of the single-set vs. multi-set approaches, such that much larger numbers of SPFs may be required to obtain agreement between these approaches.

S3 Details of EET dynamics

Complementary to the discussion in the main text, we address several aspects relating to the multiple-time scale decay observed in the EET process.

S3.1 Time scales of population transfer

Taking into account that the time evolution is purely electronic up to around $\tau_0 = \tau_{\rm el} = 2$ fs (see Sec. S3.2), a non-linear least squares fit (Levenberg-Marquardt algorithm as implemented in the SciPy library, https://scipy.org) is carried out from this time onwards. The decay of the donor population was fitted to a power series multiplied by an exponential, according to the following function,

$$\rho_D(t) = p_0[w_1(1 + b_1(t-\tau_0) + b_2(t-\tau_0)^2)e^{-(t-\tau_0)/\tau'} + (1-w_1)(w_2 e^{-(t-\tau_0)/\tau_1^{\rm EET}} + (1-w_2)e^{-(t-\tau_0)/\tau_2^{\rm EET}})] \ . \tag{S5}$$

where p_0 is the analytically known value of the population from the purely electronic evolution (see Sec. S3.2). The parameters w_1 and w_2 are weighting factors that guarantee that $\rho_D(\tau_0) = p_0$. Table S6 shows the resulting decay constants. The first two time constants,

Table S6: Time constants ($\tau', \tau_1^{\rm EET}, \tau_2^{\rm EET}$) obtained from the fit of Eq. (S5) for the four relevant temperatures.

T [K]	τ' [fs]	$\tau_1^{\rm EET}$ [fs]	$\tau_2^{\rm EET}$ [fs]
0	29.5	100.6	653
100	29.4	106.8	2301
200	29.1	105.1	2571
300	32.6	105.0	2813

τ' and $\tau_1^{\rm EET}$, are largely independent of temperature, while $\tau_2^{\rm EET}$ increases with T. The time constant τ' turns out very close to the decoherence time discussed in Sec. S.3.3, $\tau' \sim \tau_d \sim 30$ fs.

The fit procedure is generally robust and converges, but the fit was found to be ambiguous specifically regarding the $\tau_2^{\rm EET}$ value for the data at $T = 300$ K. This is presumably due to the fact that these data feature a very shallow long-time decay. As a result, multiple minima appear in the fit, which can be accessed from different initial guesses. A plausible initial guess for the $\tau_2^{\rm EET}$ parameter at $T = 300$ K was derived from the asymptotic ratio of excited-state populations at different temperatures.[2] With this initial guess, convergence was obtained

[2]In further detail, an initial guess for $\tau_2^{\rm EET}$ at $T = 300$ K was obtained by multiplying the known value

with the a value of $\tau_2^{\mathrm{EET}} = 2.8$ ps for $T = 300$ K reported in Table S6. We emphasize that this sensitive dependence on the initial guess appeared exclusively for the τ_2^{EET} parameter at T =300 K.

Fig. S6 shows the fits together with the donor state population obtained from the 2L-GMCTDH simulations for the four temperatures (upper left: 0 K, upper right: 100 K, lower left: 200 K, lower right 300 K). The fitted functions describe the data properly over the full time span for all temperatures. These time constants are reported in Figure 4 of the main text. For the time constant τ_1^{EET}, the average value $\tau_1^{\mathrm{EET}} = 108$ fs is reported in the main text.

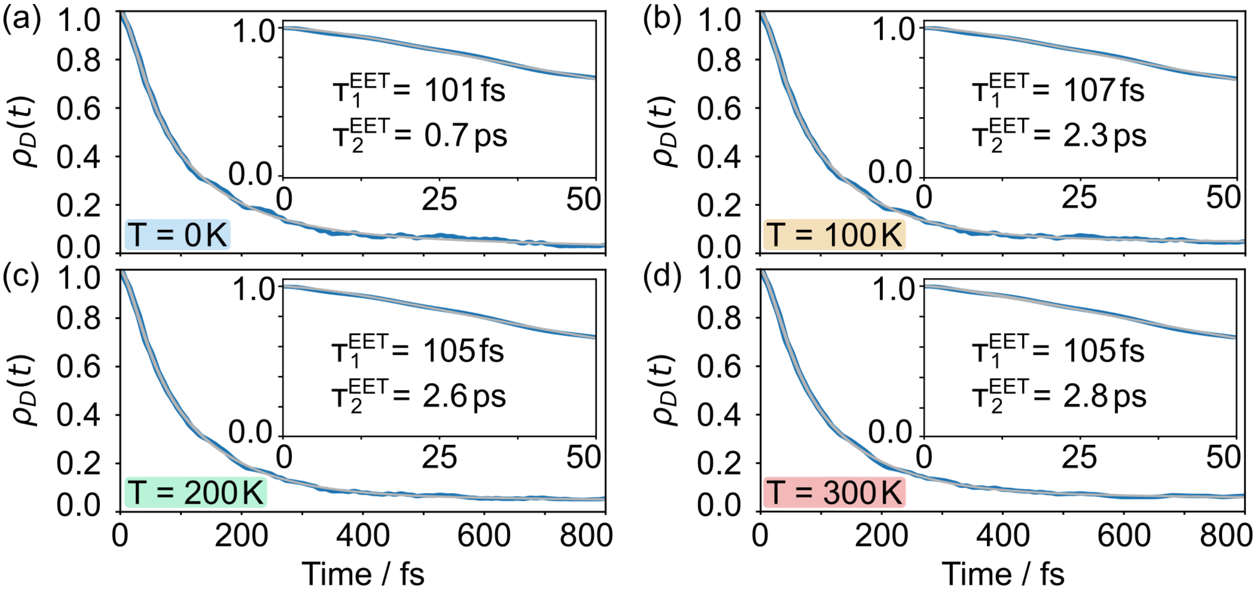

Figure S6: For $T = 0$ K, 100 K, 200 K, and 300 K, the population decay dynamics is compared with a multiple-time scale fit according to Eq. (S5). The insets show the shortest time scale up to 50 fs, which is almost independent of temperature.

of τ_2^{EET} at $T = 200$ K with the asymptotic ratio of excited-state populations at $T = 300$ K vs. $T = 200$ K, $f = 1.19$. This procedure is motivated by the fact that the ratio of τ_2^{EET} at $T = 200$ K vs. $T = 100$ K corresponds closely to the ratio of the respective excited-state populations at the final simulation time, $t = 800$ fs (here taken as the asymptotic limit).

S3.2 Initial electronic time scale

In the present system, the dynamics of the coupled electronic-vibrational system evolves from a separable initial condition defined in terms of a single electronic state (i.e., $|\mathrm{LE}^D\rangle$). System-bath correlations do not immediately build up, and an initial, very short interval therefore exists where the dynamics of the reduced electronic density matrix is determined by the purely electronic part of the Hamiltonian, $\hat{H}^{\mathrm{el}}$. Following this, decoherence sets in and a Gaussian short-time approximation for the purity decay can be made (see Sec. S3.3).

We recall that the electronic Hamiltonian reads as follows,

$$\hat{H}^{\mathrm{el}} = \Delta E|\mathrm{LE}^A\rangle\langle\mathrm{LE}^A| + \gamma_{DA}\left(|\mathrm{LE}^D\rangle\langle\mathrm{LE}^A| + |\mathrm{LE}^A\rangle\langle\mathrm{LE}^D|\right) \tag{S6}$$

with the electronic offset ΔE of the acceptor state with respect to the donor state and the electronic coupling γ_{DA}. This two-state model can be solved analytically, such as to obtain the time dependent wavefunction coefficients, $\psi(t) = c_A(t)|\mathrm{LE}^A\rangle + c_D(t)|\mathrm{LE}^D\rangle$, with

$$c_A(t) = \frac{\gamma_{DA}}{\lambda_1}A_1 e^{-i\lambda_1 t} + \frac{\gamma_{DA}}{\lambda_2}A_2 e^{-i\lambda_2 t} \quad , \quad c_D(t) = A_1 e^{-i\lambda_1 t} + A_2 e^{-i\lambda_2 t} \tag{S7}$$

where $\lambda_{1/2} = \frac{\Delta E}{2} \pm \sqrt{(\frac{\Delta E}{2})^2 + \gamma_{DA}^2}$ are the adiabatic state energies and A_1 and A_2 are determined through the initial diabatic state populations. Here, we treat the case with $c_D(0) = 1$ and $c_A(0) = 0$. For this initial condition we obtain

$$A_1 = -\frac{\lambda_1^2}{\lambda_1^2 + \gamma_{DA}^2} \quad , \quad A_2 = \frac{\gamma_{DA}^2}{\lambda_1^2 + \gamma_{DA}^2} \; . \tag{S8}$$

In Fig. S7 we compare the time evolution of the donor state population, along with the real and imaginary parts of the coherence for the pure electronic two–state system vs. the full system. We can see that the full system follows the pure electronic results for about $\tau_{\mathrm{el}} \sim 2$ fs. Beyond this purely electronic time scale, the influence of the vibrational subspace sets in. The fastest vibrational mode in our model has a period of about 20 fs, such that the 2 fs time scale corresponds roughly to one tenth of the period of this fastest mode.

S3.3 Decoherence and purity decay

As can be seen from Fig. S7, the purity of the full system remains equal to 1 during a very short initial time interval up to $\tau_0 = \tau_{\mathrm{el}} \sim 2$ fs, preceding the formation of correlations between the electronic and vibrational subsystems. After this time, we expect that a short-time Gaussian decay of the purity should hold, $\mathcal{P} \sim \exp(-((t-\tau_{\mathrm{el}})/\tau_{\mathrm{d}})^2)$,[9] where the decoherence

time is given as follows for a spin-boson model,[10,11]

$$\tau_{\mathrm{d}}^{-2} \;=\; 2\langle\delta^2\hat{S}\rangle\langle\delta^2\hat{\mathcal{E}}_{DA}^2\rangle = 2|c_D|^2|c_A|^2K_\Delta^2\langle\hat{X}_\Delta^2\rangle \tag{S9}$$

Here, $\langle\delta^2\hat{\mathcal{E}}_{DA}\rangle$ relates to the variance of energy gap fluctuations induced by the bath modes, which can be expressed in terms of the variance of an effective mode, $\langle\delta^2\hat{\mathcal{E}}_{DA}\rangle = K_\Delta^2\langle\hat{X}_\Delta^2\rangle = \sum_{s,n}\kappa_{n,s}^2\langle\hat{x}_{n,s}^2\rangle$. The effective mode $\hat{X}_\Delta$ is constructed similarly to the procedure outlined in Sec. S1.2 and represents a difference mode, i.e., $K_\Delta\hat{X}_\Delta = K_1^D\hat{X}_1^D - K_1^A\hat{X}_1^A$. Here, $\hat{X}_1^D = (K_1^D)^{-1}\sum_{n=1}^{N_D}\kappa_{n,D}\hat{x}_{n,D}$ and analogously for $\hat{X}_1^A$, as described in Sec. S1.2. When constructing the variance of the energy gap operator

$$\hat{\mathcal{E}}_{DA} = \sum_{n=1}^{N_D}\kappa_{n,D}\hat{x}_{n,D} - \left(\Delta E + \sum_{n=1}^{N_A}\kappa_{n,A}\hat{x}_{n,A})\right) \tag{S10}$$

one obtains the relation $\langle\delta^2\hat{\mathcal{E}}_{DA}\rangle = K_\Delta^2\langle\hat{X}_\Delta^2\rangle$.

Conversely, $\langle\delta^2\hat{S}\rangle = |c_D|^2|c_A|^2$ represents the subsystem variance, where c_D and c_A are the wavefunction coefficients at time τ_{el} as defined above. We note that at time $t = 0$ where $c_D(0) = 1$, the subsystem variance vanishes, i.e., $\langle\delta^2\hat{S}\rangle(0) = 0$, such that the short-time result evaluated at $t = 0$ predicts a complete absence of decoherence. Since the electronic coupling is coordinate independent in our model, the same results hold for more general short-time estimates which include contributions from the electronically off-diagonal terms.[10]

Due to the constant purity up to $\tau_0 = \tau_{\mathrm{el}}$, we can infer that system-bath correlations have not yet built up, such that the state of the system remains separable and can be represented as $|\psi\rangle(\tau_{\mathrm{el}}) = (c_D(\tau_{\mathrm{el}})|\mathrm{LE}^D\rangle + c_A(\tau_{\mathrm{el}})|\mathrm{LE}^A\rangle)\otimes\psi_{\mathrm{vib}}$. For a separable state, decoherence sets in quadratically with time,[3] and a Gaussian decay is expected according to Eq. (S9).

Fig. S8a) shows a Gaussian fit from $t = \tau_{\mathrm{el}} = 2$ fs onwards, on a 10 fs time scale where a Gaussian approximation indeed holds, resulting in a decoherence time of $\tau_{\mathrm{d}} \sim 32$ fs. This decay time is in qualitative agreement with the analytical estimate obtained from Eq. (S9) which varies between $\tau_{\mathrm{d}} \sim$ 30-50 fs, with the initial wavefunction coefficients taken between $t = \tau_0 = \tau_{\mathrm{el}} \sim$ 2-4 fs, and the variance $\langle\delta^2\hat{\mathcal{E}}_{DA}\rangle = K_\Delta^2\langle\hat{X}_\Delta^2\rangle$ taking into account the initial displacement of the vibrational modes.

In Fig. S8b), a comparison with pure-dephasing dynamics for our system is shown, in the absence of electronic coupling and identical initial occupancies of the two electronic states, i.e., $|\psi\rangle(0) = 2^{-1/2}(|\mathrm{LE}^D\rangle + |\mathrm{LE}^A\rangle)\otimes\psi_{\mathrm{vib}}$. It is seen that the purity decay in the latter case is extremely fast, with an initial Gaussian decay with decoherence time of $\tau_{\mathrm{d}} \sim 9$ fs. This is mainly due to the smaller subsystem variance $\langle\delta^2 S\rangle$ in the case where one of the wavefunction coefficients by far dominates.

[3] By computing the time derivative of the subsystem purity for factorized initial conditions and a general system-bath Hamiltonian $\hat{H} = \hat{H}_S + \hat{H}_B + \hat{H}_{SB}$, one obtains $\dot{\mathcal{P}}(t) = 2\,\mathrm{Tr}_{\mathrm{el}}\left(\dot{\hat{\rho}}^{\mathrm{el}}\hat{\rho}^{\mathrm{el}}\right) = -\frac{2i}{\hbar}\mathrm{Tr}_S\left(\mathrm{Tr}_B([\hat{H},\rho])\rho_S\right)$. Evaluation at $t = 0$ yields $\frac{d\mathcal{P}}{dt}\big|_{t=0} = 0$. Hence, the purity decay builds up quadratically in time.

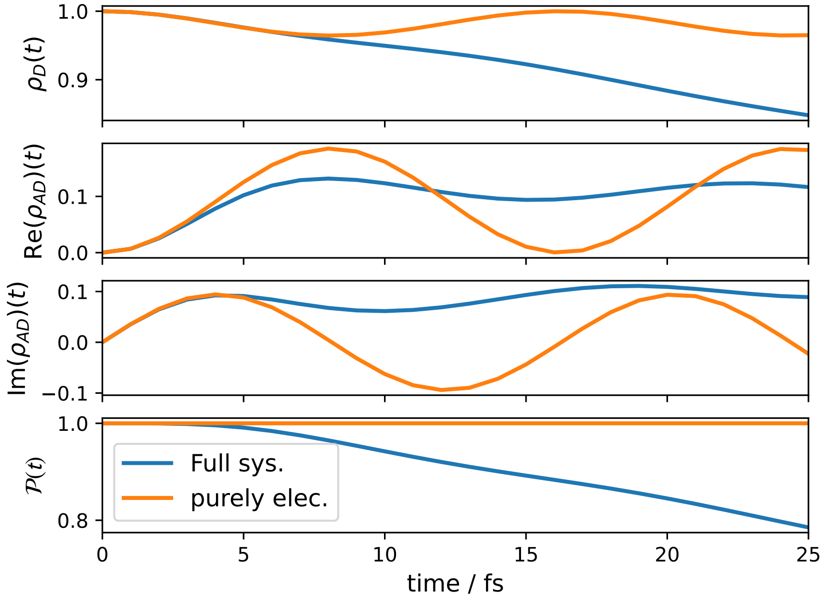

Figure S7: Evolution under the purely electronic Hamiltonian $\hat{H}^{\mathrm{el}}$ is compared with the full evolution on a 25 fs interval. It is seen that the full evolution coincides with the electronic evolution on a very short time scale around ∼2 fs.

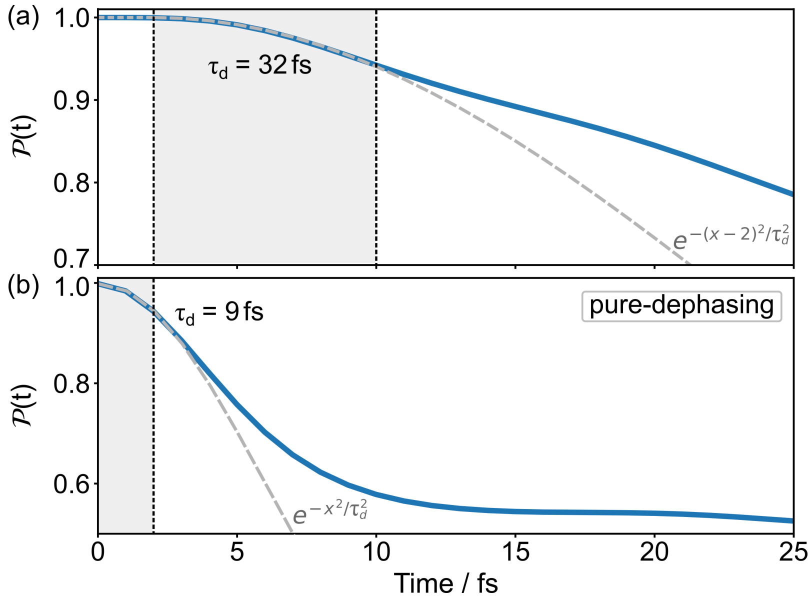

Figure S8: (a) Purity decay in our system, where a decoherence time $\tau_{\mathrm{d}} \sim 32$ fs is found beyond the purely electronic time scale $\tau_{\mathrm{el}} \sim 2$ fs (indicated by a dashed vertical line), (b) For reference, purity decay in a pure-dephasing situation, i.e., in the absence of an electronic coupling with an initial condition $|\psi\rangle(0) = 2^{-1/2}(|\mathrm{LE}^D\rangle + |\mathrm{LE}^A\rangle) \otimes \psi_{\mathrm{vib}}$. Grey areas indicate the time window where a Gaussian fit was applied.

S3.4 Energy redistribution

Complementary to the decay properties of electronic populations and coherences, we consider here the energy redistribution between electronic and vibrational energy. To this end, the time-dependent expectation values of the state-specific electronic and vibrational energies are considered. For the time-dependent effective electronic energies, we obtain

$$\langle E_s^{\mathrm{el}} \rangle(t) \;=\; \mathrm{Tr}\left\{|s\rangle\langle s|\left(\hat{H}_{\mathrm{el}} + \hat{H}_s^{\text{el-vib}}\right)\hat{\rho}(t)\right\} = \langle \hat{H}_s^{\mathrm{el}} \rangle(t) + \langle \hat{H}_s^{\text{el-vib}} \rangle(t) \tag{S11}$$

where s relates to the two states $|\mathrm{LE}^D\rangle$ and $|\mathrm{LE}^A\rangle$, $\hat{\rho}(t) = |\psi(t)\rangle\langle\psi(t)|$ is the time-evolving state, and the electronic and vibronic contributions of the Hamiltonian, $\hat{H}^{\mathrm{el}}$ and $\hat{H}^{\text{el-vib}}$, are as specified in the main text. Specifically, the state-dependent vibronic contributions are given as $\hat{H}_s^{\text{el-vib}} = \sum_{n=1}^{N_s} \kappa_{n,s}\hat{x}_{n,s}|\mathrm{LE}^s\rangle\langle\mathrm{LE}^s|$.

The state-specific vibrational energies are obtained as

$$\begin{aligned}\langle E_s^{\mathrm{vib}} \rangle(t) \;&=\; \mathrm{Tr}\left\{|s\rangle\langle s|\left(\hat{H}^{\text{el-vib}} + \hat{H}_{\mathrm{vib}}\right)\hat{\rho}(t)\right\} - E_s^{\mathrm{ZPE}} \\ &=\; P_s(t)\left(\langle \hat{H}_s^{\mathrm{vib}} \rangle(t) - E_s^{\mathrm{ZPE}}\right)\end{aligned} \tag{S12}$$

where the state-specific vibrational Hamiltonian $\hat{H}_s^{\mathrm{vib}}$ is defined with respect to the state-specific equilibrium geometry which results from the vibronic couplings,

$$\hat{H}_s^{\mathrm{vib}} \;=\; \sum_{n=1}^{N} \frac{\omega_{n,s}}{2}\left((\hat{x}_{n,s} - x_{\mathrm{eq,s}})^2 + \hat{p}_{n,s}^2\right)\hat{\mathbf{1}} \tag{S13}$$

where $x_{\mathrm{eq,s}} = -\kappa_{n,s}/\omega_n$ and all modes (N) are included, including those which feature zero displacements. The state-specific zero-point energy E_s^{ZPE} is subtracted.

S4 Vibrational displacements and occupation numbers

S4.1 State-specific mode displacements

In Figure S10, state-specific mode displacements are illustrated, i.e., displacements of mode $x_{n,s}$ in state s' (even if this mode does not couple to state s'):

$$\begin{aligned}\langle x_{n,s}^{(s')} \rangle(t) \;&=\; \mathrm{Tr}\left\{|s'\rangle\langle s'|\hat{x}_{n,s}\hat{\rho}(t)\right\}\Big/\mathrm{Tr}\left\{|s'\rangle\langle s'|\hat{\rho}(t)\right\} \\ &=\; (P_{s'}(t))^{-1}\langle\psi_{s'}(t)|\hat{x}_{n,s}|\psi_{s'}(t)\rangle\end{aligned} \tag{S14}$$

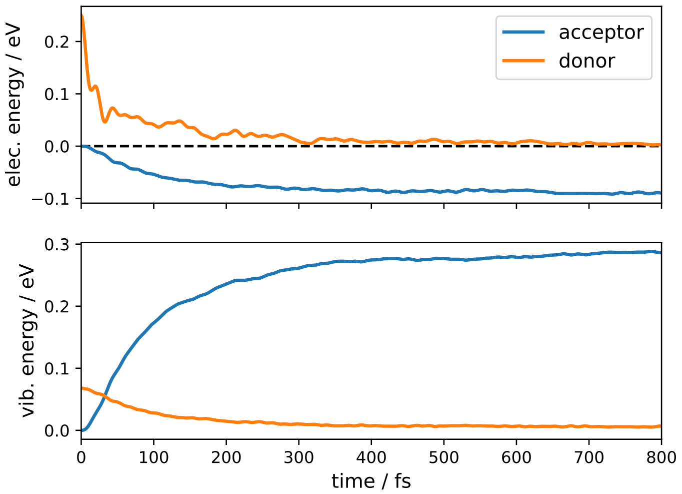

Figure S9: Time-dependent state-specific electronic and vibrational energies are shown according to Eqs. (S11)-(S12). The $|\mathrm{LE}^A\rangle$ (acceptor) state accumulates a large amount of vibrational energy, including significant contributions by vibrationally hot donor modes which are included in Eq. (S12).

Here, the multiconfigurational wavefunction of Eq. (13) in the main text is re-written as $|\Psi\rangle = \sum_s^{n_s} \psi_s |s\rangle$ for convenience. For the donor modes, significant oscillatory displacements are observed in the photoexcited $|\mathrm{LE}^D\rangle$ state (upper left panel), noting that these displacements take either positive or negative values, due to the shifted potentials. These oscillations carry over to the acceptor potential (lower left panel) where sign alternations are observed because the potentials of all donor modes are centered on zero on the acceptor potential surface. For the acceptor modes, little displacements are observed on the donor potential surface (upper right panel), but significant, constant displacements are observed on the acceptor potential surface.

S4.2 State-specific occupation numbers

Complementary to Fig. 6c-d) in the main text, state-specific mode occupation numbers are shown for all modes and states,

$$\begin{aligned} \langle \hat{N}_{n,s}^{(s')} \rangle(t) &= \mathrm{Tr}\Big\{ |s'\rangle\langle s'| \hat{N}_{n,s}^{(s')} \hat{\rho}(t) \Big\} \Big/ \mathrm{Tr}\Big\{ |s'\rangle\langle s'| \hat{\rho}(t) \Big\} \\ &= (P_{s'}(t))^{-1} \langle \psi_{s'}(t) | \hat{N}_{n,s} | \psi_{s'}(t) \rangle \end{aligned} \quad \text{(S15)}$$

where the number operator is defined for the shifted, state-specific harmonic potentials of

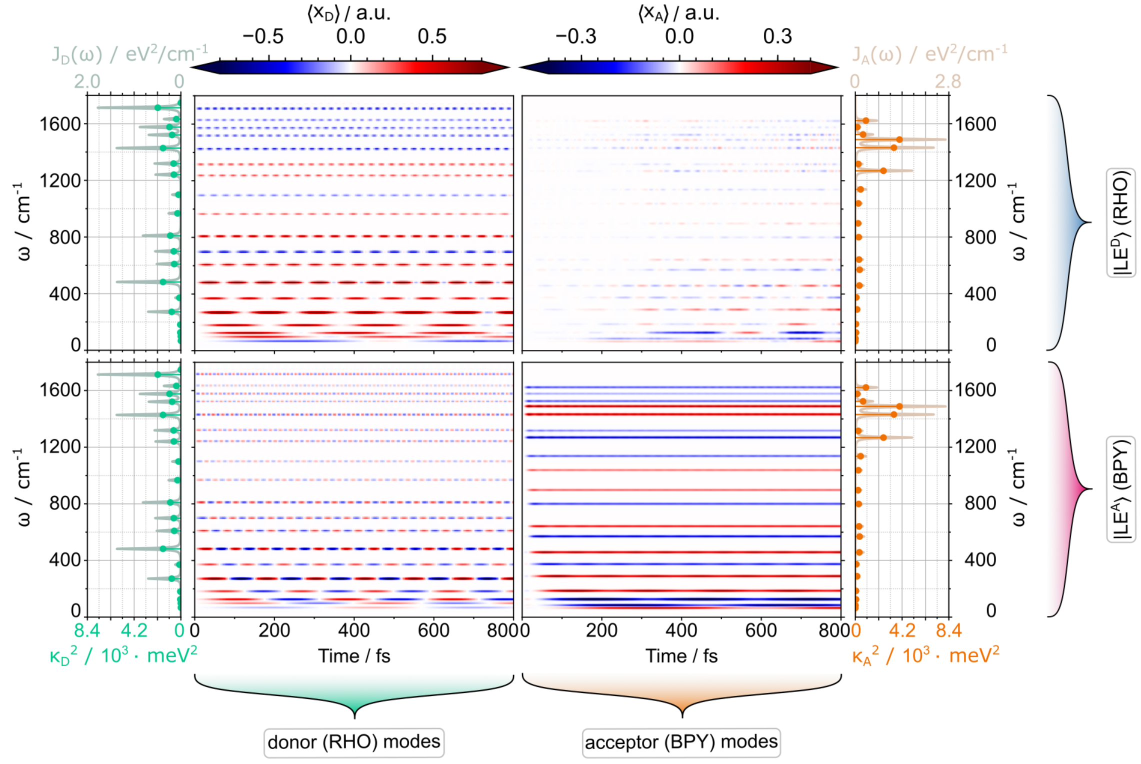

Figure S10: State-specific mode displacements are shown for the donor modes (l.h.s. panels) and the acceptor modes (r.h.s. panels). Displacements are shown for both states $|\mathrm{LE}^D\rangle$ and $|\mathrm{LE}^A\rangle$.

the selected mode:

$$\hat{N}_{n,s}^{(s')} = \frac{1}{\omega_{n,s}}\hat{h}_{n,s}^{(s')} - \frac{1}{2} \tag{S16}$$

with the state-specific vibrational Hamiltonian for mode $x_{n,s}$ in state s':

$$\hat{h}_{n,s}^{(s')} = \frac{\omega_{n,s}}{2}\left((\hat{x}_{n,s} - x_{\mathrm{eq},ns}^{(s')})^2 + \hat{p}_{n,s}^2\right) \tag{S17}$$

Here, the state-specific displacement is given as $x_{\mathrm{eq},ns}^{(s')} = -\delta_{ss'}\kappa_{n,s}/\omega_{n,s}$, that is, the displacement is zero if $s \neq s'$.

From Fig. S11, it is seen that several modes exhibit significant vibrational excitation, especially donor modes which remain vibrationally hot in the $|\mathrm{LE}^A\rangle$ state. Specifically, the donor modes at 272 cm^{-1} and 482 cm^{-1} play a significant role (see Fig. 6 in the main text). Among the acceptor modes, mode-specific energy absorption is much less pronounced, but several high-frequency modes (> 1200 cm^{-1}) exhibit larger occupation numbers.

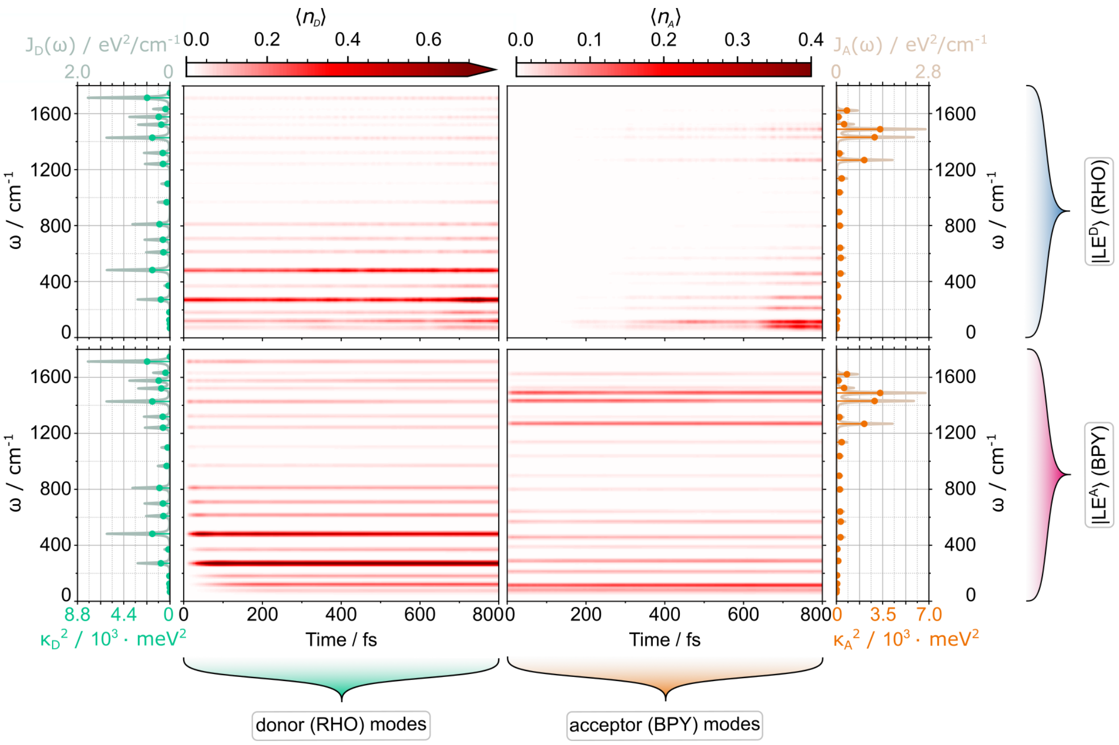

Figure S11: State-specific mean occupation numbers are shown for the donor modes (l.h.s. panels) and the acceptor modes (r.h.s. panels). Occupation numbers are shown for both states $|\mathrm{LE}^D\rangle$ and $|\mathrm{LE}^A\rangle$. Note the different scales for the occupation numbers associated with the $|\mathrm{LE}^D\rangle$ and $|\mathrm{LE}^A\rangle$ states.

S5 Mode specificity and vibronic resonance effects

S5.1 Fourier analysis of coherence evolution

As can be seen from Fig. 4b-c) in the main text, the electronic coherence shows a pronounced oscillatory behavior. These oscillations appear more complex than just Rabi oscillations between the two electronic states, and it is to be expected that vibronic components appear. To analyse the frequency components, a Fourier transform of $\mathrm{Re}\rho_{DA}$ and $\mathrm{Im}\rho_{DA}$ was carried out, illustrated in Fig. S12. Given that the electronic coupling γ_{DA} is comparatively small (0.024 eV), the evolution of the electronic coherence is going to involve difference frequencies between vibronic levels pertaining to the two diabatic states. If this difference corresponds to the electronic energy gap, a vibronic resonance effect can be sustained.

Focussing on the oscillatory behavior beyond the initial decay, we subtracted the initial decay by fitting an exponential function independently to both $\mathrm{Re}\rho_{DA}$ and $\mathrm{Im}\rho_{DA}$, and conducted a spectral analysis on the remaining residuals. The resulting power spectrum is shown in Fig. S12. The spectrum was calculated by means of the maximum entropy method of Burg[12] as implemented in Ref. [13]. The power spectrum shows several distinct peaks that are not simply harmonics or subharmonics of the electronic oscillations which are well

approximated by the energy gap, $|\Delta E| = 0.25$ eV (i.e., $\omega_{\rm el} = 2016.4$ cm^{-1} corresponding to a periodicity of 16.5 fs). Instead, multiple peaks are observed which correspond to vibronic transitions. The lowest visible difference frequency relates to a combination of two modes, namely $M_5^D + M_{19}^D$ (or $M_7^D + M_{16}^D$) whose frequencies sum up to nearly 0.25 eV, i.e., the value of the purely electronic energy gap. Besides the modes M_5^D (272 cm^{-1}) and M_7^D (482 cm^{-1}) and the complementary high-frequency modes M_{16}^D (1521 cm^{-1}) and M_{19}^D (1713 cm^{-1}), other modes or combinations of modes with comparatively large vibronic couplings participate.

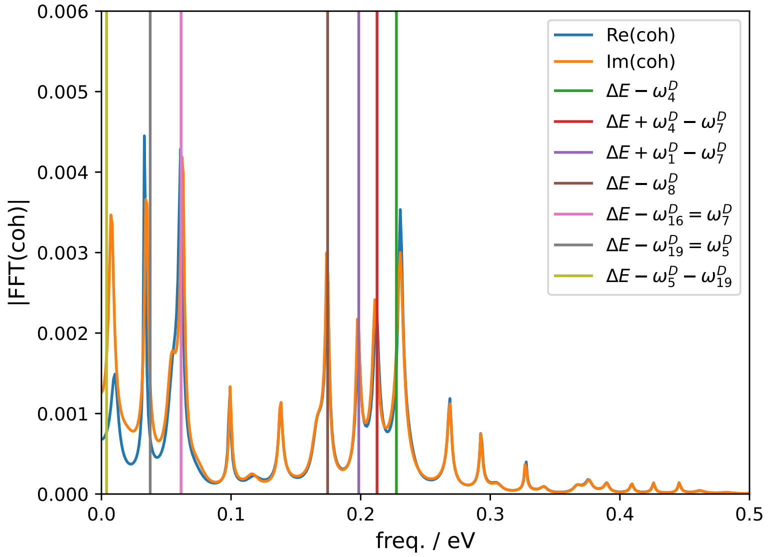

Figure S12: Fourier transform of the real and imaginary parts of the electronic coherence, Reρ_{DA} (blue) and Imρ_{DA} (orange). The vertical lines indicate selected frequency components appearing in the time evolution.

S5.2 Exclusion of subsets of modes from the dynamics

Since specific modes play a prominent role in the dynamics and these belong to the low to intermediate frequency range, we carried out simulations where modes below 1000 cm^{-1} were excluded from the dynamics. As can be seen from the orange trace in Fig. S13, this entirely blocks the EET dynamics. Conversely, switching off all modes above 1000 cm^{-1} (red trace) reduces the transfer efficiency, but does not completely impede the transfer. Interestingly, preserving only modes M_5^D (272 cm^{-1}) and M_7^D (482 cm^{-1}) among the low-frequency modes (in addition to the high-frequency modes) leads to a transfer profile that is quite close to the full EET dynamics (see green trace as compared with blue trace).

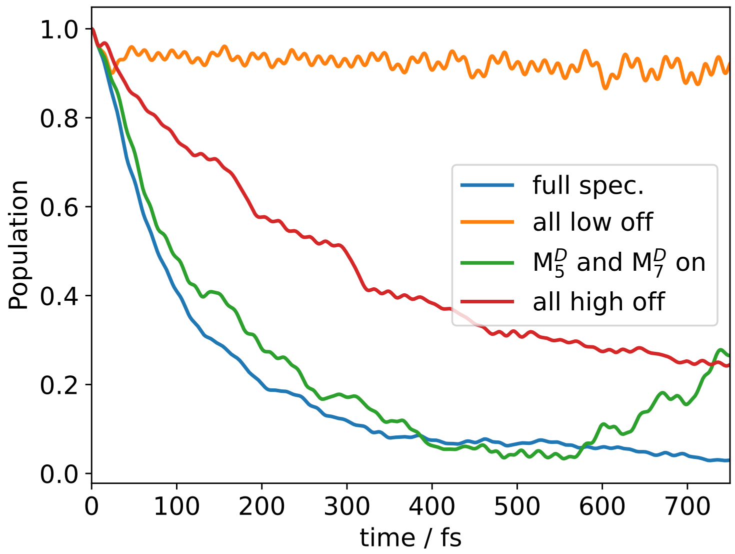

Figure S13: EET dynamics involving different subsets of modes, as compared with the reference calculation (blue): only high-frequency modes > 1000 cm^{-1} (orange), only intermediate- and low-frequency modes < 1000 cm^{-1} (red), high-frequency modes > 1000 cm^{-1} plus modes M_5^D (272 cm^{-1}) and M_7^D (482 cm^{-1}) (green).

S6 Reduced two-effective mode dynamics

Complementary to Fig. 7 in the main text, the EET dynamics of a minimal model comprising two vibrational modes is addressed. Given that the donor vibrational modes play a dominant role in the process, the first two effective modes resulting from the transformation of the donor spectral density (see Sec. S1) were considered, with frequencies $\omega_1^D = 544$ cm^{-1} and $\omega_2^D = 1493$ cm^{-1}.

The LVC Hamiltonian for this system is given as follows, $\hat{H}^{\text{LVC}} = \hat{H}^{\text{el}} + \hat{H}^{\text{vib}} + \hat{H}^{\text{el-vib}}$, where the electronic part is unchanged as compared with the full model,

$$\hat{H}^{\text{el}} = \Delta E|\text{LE}^A\rangle\langle\text{LE}^A| + \gamma_{DA}\left(|\text{LE}^D\rangle\langle\text{LE}^A| + c.c.\right) \tag{S18}$$

where $\Delta E = -0.25$ eV is the donor-acceptor offset, and $\gamma_{DA} = 0.024$ eV is the EET coupling between the two locally excited (LE) states. The vibrational and vibrational-electronic (vibronic) parts of the Hamiltonian are given as follows,

$$\hat{H}^{\text{vib}} = \sum_{n=1}^{2} \frac{\omega_{n,D}}{2}(\hat{x}_{n,D}^2 + \hat{p}_{n,D}^2)\mathbf{1} \tag{S19}$$

and

$$\hat{H}^{\text{el-vib}} = \sum_{n=1}^{2} \kappa_{n,D}\hat{x}_{n,D}|\text{LE}^D\rangle\langle\text{LE}^D| \tag{S20}$$

where $\kappa_{1,D} = 0.097$ eV and $\kappa_{2,D} = 0.203$ eV.

The potential crossing is defined by the condition $0 = \Delta E - \kappa_{1,D}x_{1,D} - \kappa_{2,D}x_{2,D}$, that is,

$$x_{2,D} = \frac{\Delta E - \kappa_{1,D}x_{1,D}}{\kappa_{2,D}} \tag{S21}$$

This condition defines the diabatic seam line shown in Figure 7 in the main text. The minimum energy point along the diabatic seam is given at the following coordinate values,

$$x_{1,D}^{\text{min}} = \frac{\omega_{2,D}\kappa_{1,D}\Delta E}{\omega_{1,D}\kappa_{2,D} + \omega_{2,D}\kappa_{1,D}} \quad , \quad x_{2,D}^{\text{min}} = \frac{\Delta E - \kappa_{1,D}x_{1,D}^{\text{min}}}{\kappa_{2,D}}$$

S6.1 Population decay, coherence, purity

Complementary to Fig. 4 in the main text, Fig. S14 shows the population dynamics, coherence, and purity for the reduced 2-mode system as compared with the full dynamics, on a time scale of 200 fs. While this approximation is expected to be exact on a very short time scale, determined by the first few cumulants of the propagator,[14] it is seen that qualitative agreement is obtained on the longer time scale shown here. The initial decay dynamics characterized by the vibronic resonance effects discussed in the main text, is well reproduced on a time scale up to ∼100 fs.

S6.2 Mode displacements and occupation numbers

In Fig. S15, the time-dependent displacements and occupation numbers of the two modes are shown. While both modes exhibit regular oscillations, the low-frequency mode (M_1) shows occupation numbers up to around 2.5 in the $|\text{LE}^A\rangle$ state, while occupation numbers of the high-frequency mode (M_2) remain below 1. This reflects that the M_1 mode accepts a significant amount of energy through a vibronic resonance effect as discussed in the main text.

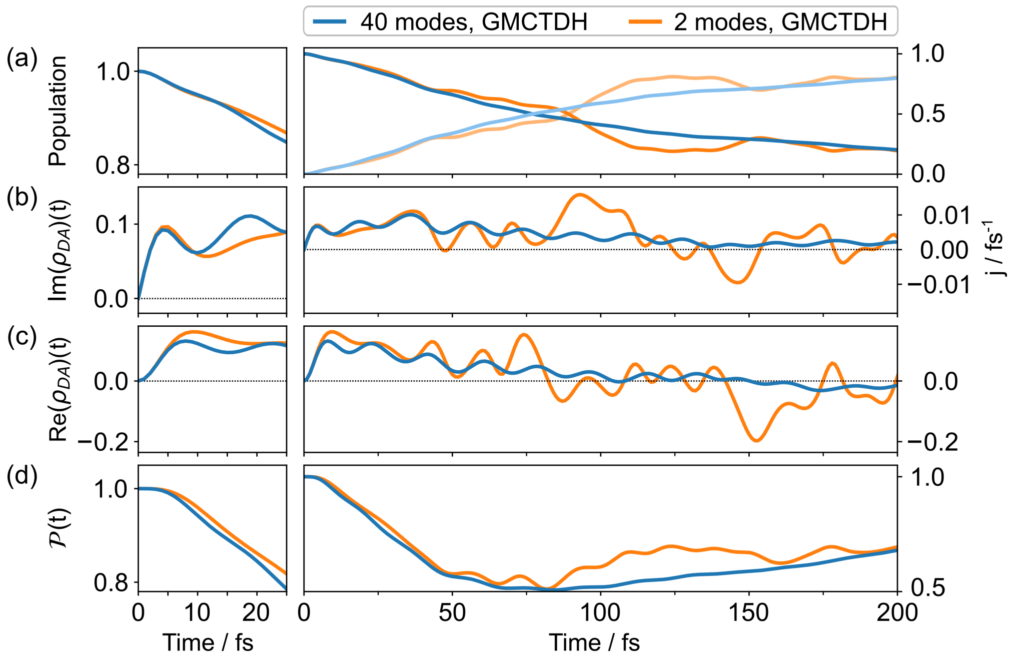

Figure S14: Analogously to Fig. 4 in the main text, 2L-GMCTDH calculations are shown for a) the state populations, b-c) the real and imaginary parts of the electronic coherence, d) the purity is shown for the reduced two-mode model (orange lines) as compared with the full 40-mode dynamics (blue lines) at $T = 0$K.

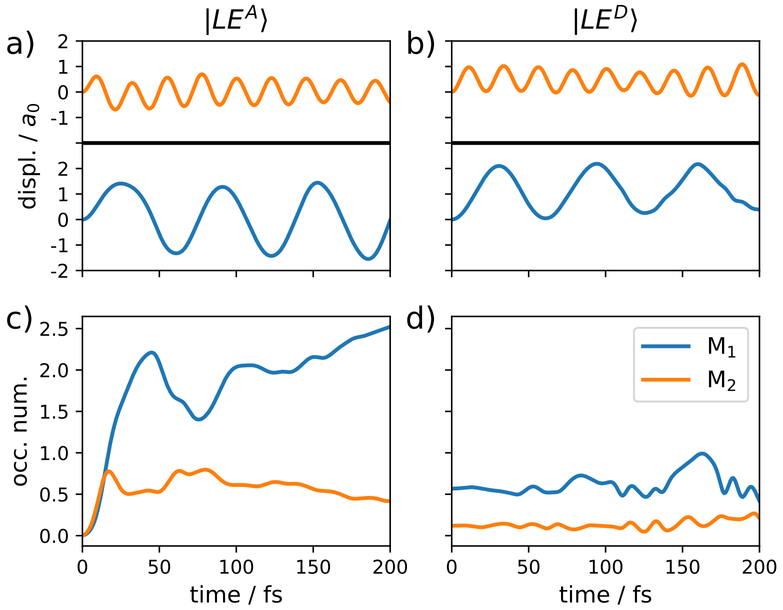

Figure S15: a)-b) State-specific displacements and c)-d) state-specific occupation numbers are shown for the two-mode model.

S6.3 2D densities in comparison with MCTDH

In Fig. S16, time-evolving state-specific mode densities are shown for the minimal 2-mode system within the first 90 fs. In addition to multi-set 2L-GMCTDH calculations, multi-set MCTDH reference calculations are illustrated, showing very good agreement. In the multi-set MCTDH calculations, two one-dimensional particles were used per state, each with 5 SPFs per state, while the 2L-GMCTDH calculation used a combined first-layer particle with 3 SPFs per state, with two second-layer GWP particles, with 4 GWPs per state.

The densities in the acceptor state $|\mathrm{LE}^A\rangle$ exhibit significant complexity, with a node pattern that emerges within the first 30 fs, due to the vibronic resonance effects that are discussed in the main text.

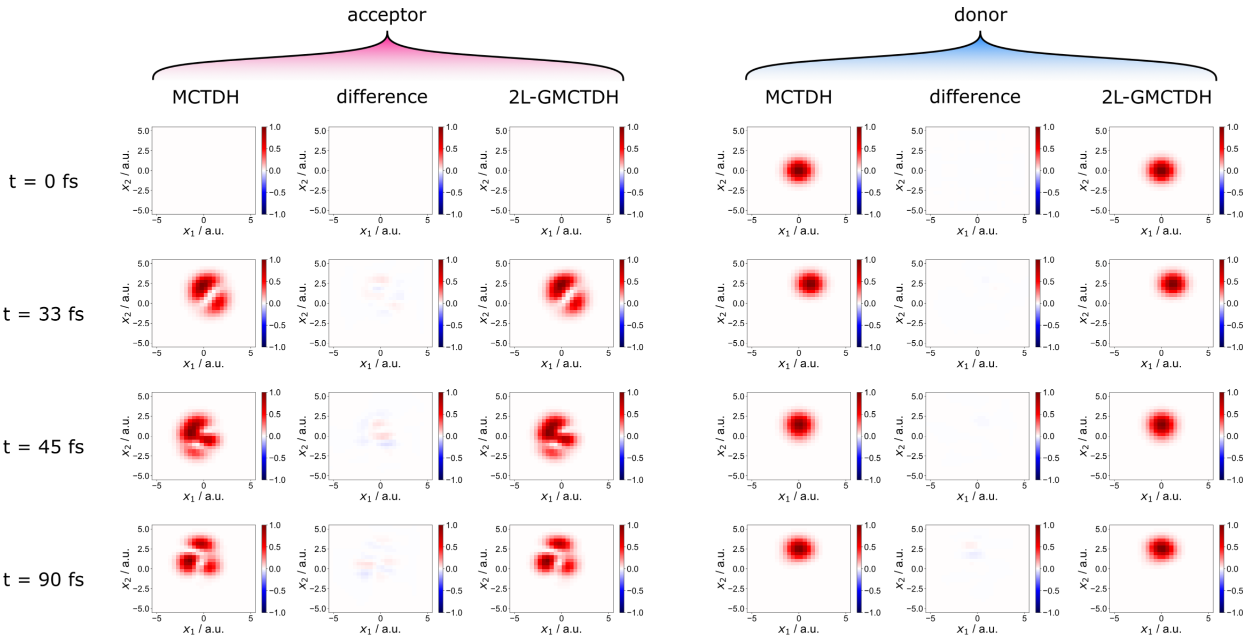

Figure S16: Snapshots of the time-evolving densities are shown for the minimal 2-mode system in the interval 0-90 fs. Multi-set 2L-GMCTDH calculations are compared with multi-set MCTDH reference calculations, showing very good agreement.

S7 State-to-state flux

As in the Appendix, we consider the continuity equation for the subsystem (electronic) density matrix. However, we generalize Eq. (A.2) to the case of a generic Hamiltonian, $\hat{H} = \sum_{ss'} H_{ss'}|s\rangle\langle s'|$ which can include a dependence on the bath coordinates and which

could also relate to external fields. Analogously to Eq. (A.2), we obtain

$$\begin{aligned}\frac{d\rho^{\mathrm{el}}_{ss}}{dt} &= -i\,\underset{\mathrm{vib}}{\mathrm{Tr}}\left\langle s\left|\left[\hat{H},\hat{\rho}(t)\right]\right|s\right\rangle \\ &= -i\,\underset{\mathrm{vib}}{\mathrm{Tr}}\sum_{s'\neq s}\langle s\,|(H_{ss'}|s\rangle\langle s'|\hat{\rho}-\hat{\rho}|s'\rangle\langle s|H_{s's}]|\,s\rangle \\ &= -i\,\underset{\mathrm{vib}}{\mathrm{Tr}}\sum_{s'\neq s}(H_{ss'}\rho_{s's}-\rho_{ss'}H_{s's}) \\ &= \underset{\mathrm{vib}}{\mathrm{Tr}}\sum_{s'\neq s}(-i\,\mathrm{Re}(\rho_{s's})\,(H_{ss'}-H_{s's})+\mathrm{Im}(\rho_{s's})\,(H_{ss'}+H_{s's})) \\ &\equiv \sum_{s'} j_{s's}(t) \qquad \text{(S22)}\end{aligned}$$

If the Hamiltonian is time reversal invariant, it features real-valued elements $H_{ss'} = H_{s's}$ such that the first term on the r.h.s. of the penultimate line disappears while the second term yields $j_{s's}(t) = 2\,\underset{\mathrm{vib}}{\mathrm{Tr}}\{\,H_{s's}\mathrm{Im}(\rho_{s's})\,\}$. For a coordinate independent electronic coupling term, the above expression further simplifies, leading to Eq. (A.2).

References

(1) Asido, M.; Hamerla, C.; Weber, R.; Horz, M.; Niraghatam, M. S.; Heckel, A.; Burghardt, I.; Wachtveitl, J. Ultrafast and efficient energy transfer in a one- and two-photon sensitized rhodamine-BODIPY dyad: a perspective for broadly absorbing photocages. *Phys. Chem. Chem. Phys.* **2022**, *24*, 1795–1802.

(2) Hughes, K. H.; Christ, C. D.; Burghardt, I. Effective-mode representation of non-Markovian dynamics: A hierarchical approximation of the spectral density. I. Application to single surface dynamics. *J. Chem. Phys.* **2009**, *131*, 024109.

(3) Hughes, K. H.; Christ, C. D.; Burghardt, I. Effective-mode representation of non-Markovian dynamics: A hierarchical approximation of the spectral density. II. Application to environment-induced nonadiabatic dynamics. *The Journal of Chemical Physics* **2009**, *131*, 124108.

(4) Popp, W.; Polkehn, M.; Hughes, K. H.; Martinazzo, R.; Burghardt, I. Vibronic coupling models for donor-acceptor aggregates using an effective-mode scheme: Application to mixed

Frenkel and charge-transfer excitons in oligothiophene aggregates. *J. Chem. Phys.* **2019**, *150*, 244114.

(5) Löwdin, P.-O. On the Non-Orthogonality Problem Connected with the Use of Atomic Wave Functions in the Theory of Molecules and Crystals. *The Journal of Chemical Physics* **1950**, *18*, 365–375.

(6) Eisenbrandt, P.; Ruckenbauer, M.; Burghardt, I. Gaussian-Based Multiconfiguration Time-Dependent Hartree: A Two-Layer Approach. III. Application to Nonadiabatic Dynamics in a Charge Transfer Complex. *J. Chem. Phys.* **2018**, *149*, 174102.

(7) Eisenbrandt, P.; Ruckenbauer, M.; Römer, S.; Burghardt, I. Gaussian-Based Multiconfiguration Time-Dependent Hartree: A Two-Layer Approach. II. Application to Vibrational Energy Transport in a Molecular Chain. *J. Chem. Phys.* **2018**, *149*, 174101.

(8) Wang, H. Multilayer Multiconfiguration Time-Dependent Hartree Theory. *J. Phys. Chem. A* **2015**, *119*, 7951–7965.

(9) Schlosshauer, M. *Decoherence and the Quantum-To-Classical Transition*, 3rd ed.; Springer-Verlag, 2007.

(10) Gu, B.; Franco, I. Generalized Theory for the Timescale of Molecular Electronic Decoherence in the Condensed Phase. *J. Phys. Chem. Lett.* **2018**, *9*, 773–778.

(11) Gu, B.; Franco, I. Quantifying Early Time Quantum Decoherence Dynamics through Fluctuations. *J. Phys. Chem. Lett.* **2017**, *8*, 4289–4294.

(12) Burg, J. P. *Maximum Entropy Spectral Analysis*; Stanford University, 1975.

(13) Press, W. H. *Numerical Recipes 3rd Edition: The Art of Scientific Computing*; Cambridge University Press, 2007.

(14) Burghardt, I.; Hughes, K. H.; Martinazzo, R.; Tamura, H.; Gindensperger, E.; Köppel, H.; Cederbaum, L. S. In *Conical Intersections*; Domcke, W., Yarkony, D. R., Köppel, H., Eds.; WORLD SCIENTIFIC, 2011; pp 301–346.
